# The performance of three novel Gemini surfactants as inhibitors for acid steel corrosion: experimental and theoretical studies†

**DOI:** 10.1039/d1ra07449k

**Published:** 2021-11-22

**Authors:** Mohamed Deef Allah, Samar Abdelhamed, Kamal A. Soliman, Mona A. El-Etre

**Affiliations:** Basic Science Department, Faculty of Engineering, Benha University Shoubra Egypt hamoaaa2002@yahoo.com eng.samar843199@yahoo.com; Chemistry Department, Faculty of Science, Benha University Benha Egypt kamalsoliman@gmail.com; Basic Science Department, Faculty of Engineering, Benha University Benha Egypt monaeletr@yahoo.com

## Abstract

Adipic acid was used to synthesize three nonionic Gemini surfactants containing different numbers of propylene oxide units in their structures. The produced surfactants have been characterized employing FTIR and 1H-NMR spectra. Some of the physical properties of them, namely, surface tension, maximum surface excess concentration, surface pressure, critical micelle concentration, and the minimal area of the surface taken by a single molecule, were computed. The inhibitory effect of the synthesized surfactants on the corrosion of C-steel (C45) in 1.0 M HCl solution was studied. Gravimetric and electrochemical methods were used for corrosion rate measurements. The outcomes acquired from the used methods showed that every one of the three surfactants works as a strong inhibitor for steel acidic corrosion. By raising surfactant concentration and exposure time, the inhibition proficiency improves. The inhibition efficiency exceeded 90% for the three compounds. The higher the propylene oxide units contained in the surfactant molecule the higher is its inhibition efficiency. Based on the findings, a mechanism for inhibitory action was proposed. Moreover, the density functional theory (DFT) and molecular electrostatic potential (MEP) were investigated for the three inhibitors. The calculated parameters were correlated with the inhibition efficiency.

## Abstract 2

Adipic acid was used to synthesize three nonionic Gemini surfactants containing different numbers of propylene oxide units in their structures.

## Graphical abstract

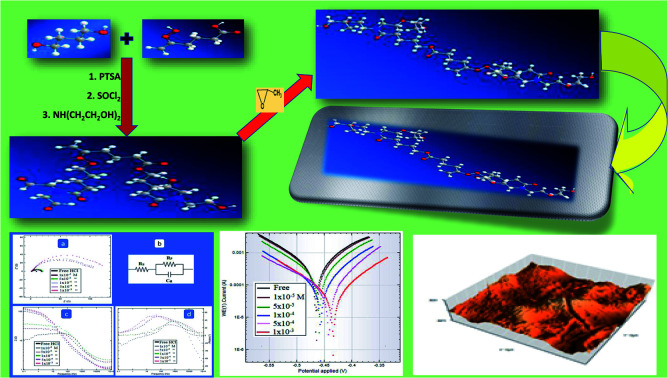

## 1. Introduction

Corrosion represents a serious problem due to its significant negative impact on the economy, especially in industrial applications.^1–5^ The corrosion impact is not only on the metals and alloys that are corroded but extends to the loss of valuable materials contained in them. A clear example is the loss of petroleum from the corroded pipelines. However, it is well known that the corrosion phenomenon is spontaneous and cannot be completely stopped. Thus, a huge number of research studies are continuously conducted to reduce the effect of corrosion to its minimum.^6–21^ Many scenarios were suggested to achieve this goal among them using corrosion inhibitors. It was found that the use of some chemical compounds had a specific effect on the original solution properties. The main characteristic of these compounds is their capability to absorb on the metal surface because of their surface properties or possession of a lone pair of electrons besides other structural properties.

Carbon steel is considered a usual choice for many industrial applications. In the petroleum industry, for example, it is used in pipelines and tanks. Although stainless steel has higher corrosion resistance than carbon steel, the latter is often preferred due to its lower cost. However, scales, as well as corrosion products, are deposited at the steel surface during operation periods. The acid-pickling process is applied to remove the scales and restore the clean surface. A corrosion inhibitor must be employed in the procedure to prevent the acid from damaging the steel surface.^22–28^

Surfactant compounds tend to accumulate with high concentrations at the interfaces. Such property introduces this category of compounds as potential corrosion inhibitors. The special type of surface-active compounds is the Gemini surfactants with their unique structure that differs from that of other surfactants. The presence of many hydrophilic as well as hydrophobic groups improves their surface properties and thus promotes their role as corrosion inhibitors. Therefore, many studies were concentrated lately on the potential use of these compounds for corrosion inhibition.^27,29–41^ Quantum calculations based on the density functional theory are used to know the mechanism between the inhibitor and the metal surface. The efficiency of the inhibitor depends mainly on the electronic properties of the inhibitor.^42–47^

The goal of this research is to create novel Gemini surfactants that can be tested as corrosion inhibitors. The study also looks at how molecular structure affects their inhibitory power. Thus, three novel Gemini surfactants based on adipic acid with different numbers of propylene oxide units were synthesized. Their structures were characterized by NMR and IR spectra. Chemical and electrochemical techniques were used to investigate their impact on carbon steel acid corrosion. The inhibitory power and molecular structures of the studied surfactants were correlated using quantum chemical calculations.

## 2. Experimental techniques

### 2.1 Materials

Across Chemical Company (UK) was used to obtain adipic acid. Sigma-Aldrich (Germany) was used to obtain 1,4 butanediol, diethanolamine, and propylene oxide. Thionyl chloride and *P*-toluene sulfonic acid were from Fluka Chemika (Germany).

Benzene, xylene as well as potassium hydroxide were brought from (Al Gomhuria Trade Pharmaceuticals & Chemical Company, Egypt).

#### 2.1.1 Synthesis of 5-{[6-(4-carboxybutoxy)-6-oxohexanoyl]oxy}pentanoic acid

Diester compound 5-{[6-(4-carboxybutoxy)-6-oxohexanoyl] oxy} pentanoic acid has been prepared *via* refluxing a mixture of 1,4 butanediol (0.05 mol) with adipic acid (0.1 mol) respectively, in 50 ml of dry benzene for six hours, in the presence of 0.1 wt% *p*-toluenesulfonic acid as a catalyst. Distilled water was added once the reaction mixture had cooled. The benzene layer which contains the diester was separated. To extract the crude product, the ester solution was vacuum distilled after being dried overnight. Fractional distillation under vacuum was used to further purify the product.^48,49^

#### 2.1.2 Synthesis of 1,6-bis(5-chloro-5-oxopentyl)hexanedioate

To a cold thionyl-chloride (10 ml) at 0 °C was added 5-{[6-(4-carboxybutoxy)-6 oxohexanoyl]oxy}pentanoic acid (5.0 g, 24.72 mmol) portion-wise into the reaction flask with continuous stirring. The temperature was gradually raised to room temperature once the addition was completed, and the stirring was continued overnight. The resultant acid chloride was then taken in 50 ml CH_2_Cl_2_ then cooled to 0 °C after the volatile was expelled under vacuum.^50^

#### 2.1.3 Synthesis of 1,6-bis({4-[bis(2-hydroxyethyl)carbamoyl]butyl})hexanedioate

In 50 ml xylene, the diester dichloro compound; 1,6-bis(5-chloro-5-oxopentyl)hexanedioate interacted with diethanolamine at a reaction molar ratio of 0.5 : 1.0, introducing dropwise triethylamine (TEA) as a catalyst while refluxing for five hours at 120 °C. Petroleum ether was being used to extract the product.^51^

#### 2.1.4 Synthesis of nonionic Gemini surfactants

**Fig. 1:**
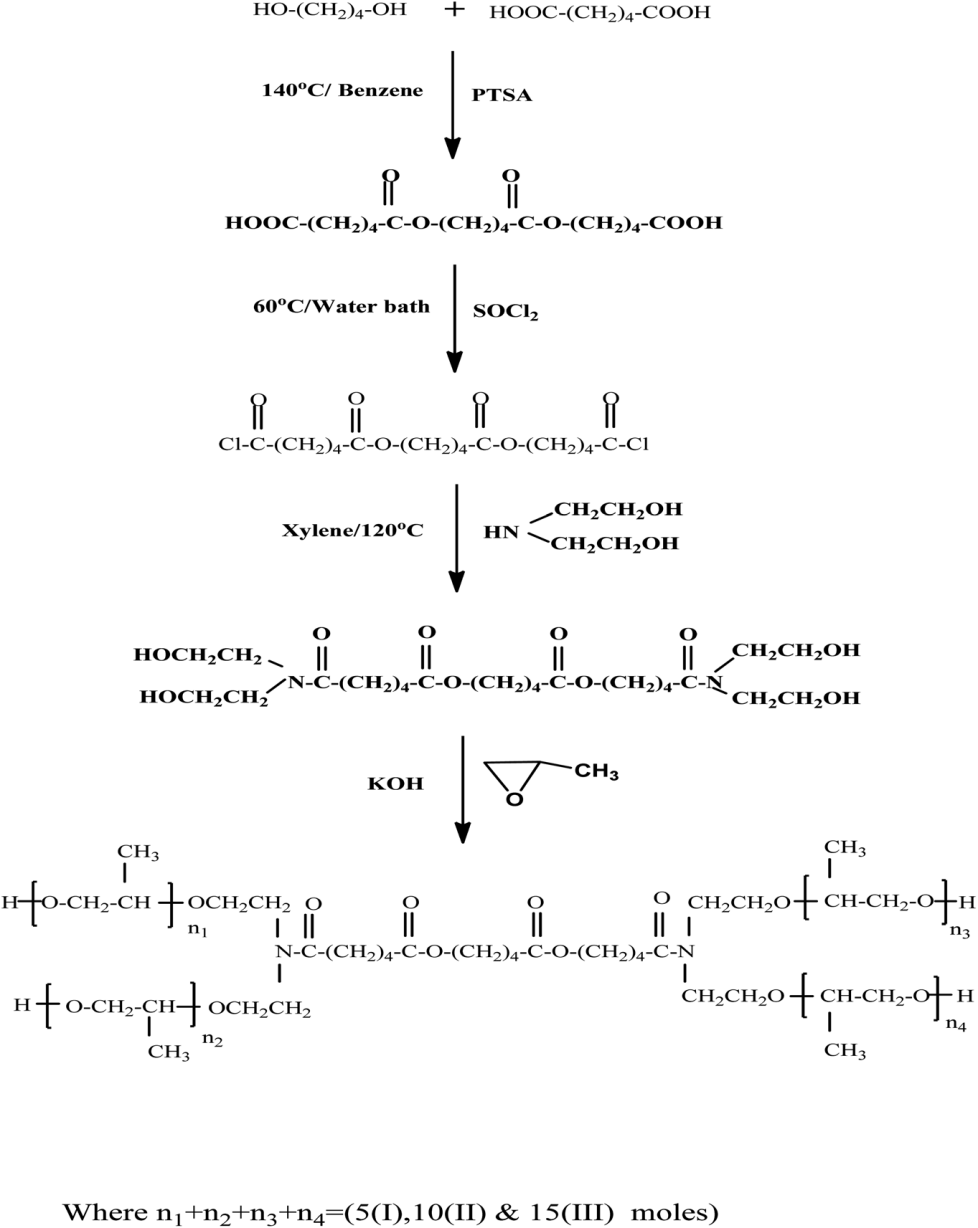
Synthesis procedure of nonionic Gemini surfactants.

To remove oxygen from the system, 0.5 wt% KOH solutions containing 0.01 mol of the prepared (diester diamino compound) generated in the third step were heated to 70 °C and stirred whilst a constant flow of nitrogen is passed into the system. The nitrogen flow was turned off, and propylene oxide was supplied in droplets with constant stirring while heated under an effective reflux system to retain the propylene oxide. The reaction was carried out at various periods varying from one to ten hours. The reaction vessel was weighed after the apparatus was cooled and saturated with nitrogen. From growth in the reaction mixture mass, the amount of propylene oxide that has been reacted as well as the average degree of propoxylation was calculated.^49^ The surfactants I, II and II were obtained with molar ratios of 5, 10, and 15, respectively (Fig. 1).

### 2.2 Physical properties

#### 2.2.1 Surface tension (*γ*)

The surface parameters of aqueous solutions of surfactants I, II & III were studied at 25 °C. The values of critical micelle concentration (CMC) were determined *via* surface tension measuring employing a tensiometer K6 device (Krüss Company, Germany) with ring method measurements (±0.2 mN m^−1^). To establish equilibrium, the solution was kept for 30 min before measuring. Each measurement was carried out three times, with a 5 minutes interval between each measurement, and the average was obtained and used for subsequent studies.^52^

#### 2.2.2 Critical micelle concentration (CMC)

The inflection points in the *γ versus* [log *C*] graphs (with of uncertainty 0.05–0.1 mM) matched to the CMCs correspond to the aqueous solutions of compounds I, II, and III at 25 °C.^53^

### 2.3 Corrosion study

#### 2.3.1 Weight-loss

Titration against a standard solution of Na_2_CO_3_ was used to adjust the concentration of HCl carbon steel (C45) with composition of: (C 0.42–0.50%, P 0.045% max, Mn 0.50–0.80%, S 0.045% max and Si 0.15–0.40%) was used in the present study. Rectangular coupons of carbon steel with dimensions of (1 cm, 1 cm, 0.1 cm) were used in the gravimetric measurements. Coupons that were examined were abraded to a mirror shine using different grades of emery papers, rinsed with distilled water followed by acetone then inserted in the test solution. Every experiment has been conducted three times and the average value was recorded. The inhibition efficacy (*η*%) and the proportion of surface area that is covered by surfactants molecules (*θ*) have been calculated the usage of the subsequent equations, respectively:1
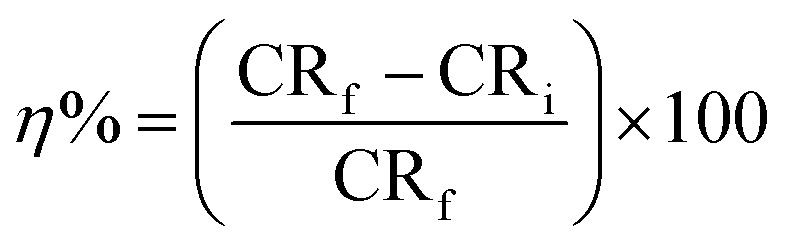
2
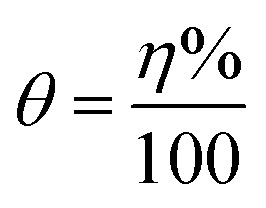
where CR_f_ and CR_i_ stand respectively for corrosion rate (g cm^−1^ h^−1^) in the uninhibited and inhibitor-containing solutions.

#### 2.3.2 Electrochemical polarization

A three-electrodes cell was used for conducting the electrochemical testing. A foil of platinum was a counter electrode while the reference one is a saturated calomel electrode. A rode of carbon steel became buried in a glass tube with Araldite, leaving an 0.1 cm^2^ backside facet uncovered to contact the corrosive medium was the working electrode. The working electrode naked tip was abraded utilizing emery papers with different grades, cleaned with distilled water, followed by acetone, dried using filter paper, and then inserted into the test solution. Before the measurements began, the steel electrode was kept in the tested solution for 10 min till it reached the value of its steady-state potential. At 25 °C, the corrosion parameters were monitored using a Metrohm potentiostat and Nova software at a 2.0 mVs^−1^ scan rate. The inhibition efficacy (*η*%) was calculated usage of the subsequent equation:3
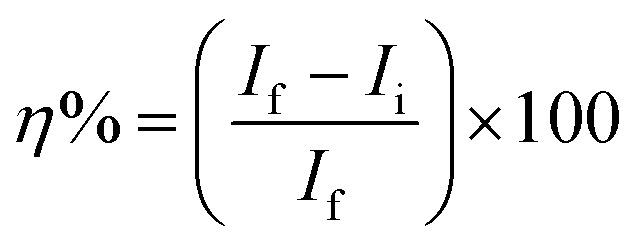
where, *I*_f_ and *I*_i_, stand for corrosion current density of the uninhibited and inhibited solutions.

Electrochemical impedance spectroscopy was applied on C-steel, in free HCl solution (1.0 M), and those inhibited by specific concentrations of the three tested surfactants. The technique was running with alternative current signals of 10 mV peak to peak amplitude over a range of frequency 10^5^ Hz to 0.1 Hz. Both Nyquist and Bode spectra have been registered using Nova software associated with the Metrohm potentiostat. In Nyquist representation, the polarization resistance (*R*_P_) was derived from the diameter of the semicircle.

The inhibition efficacy (*η*%) was figured from polarization resistance (*R*_P_) values the use of the subsequent equation:4
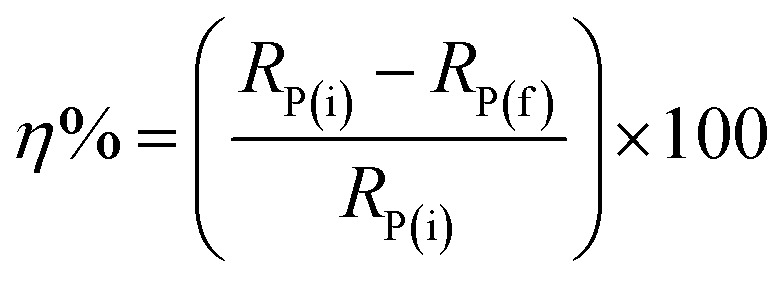


*R*_P(f)_ & *R*_P(i)_ stand respectively for polarization resistance of free and inhibited solutions.

### 2.4 Surface scanning

Using atomic force microscopy (AFM; Pico SPM-Picoscan 2100, Molecular Imaging, Arizona, AZ, USA), the carbon steel surface morphology was investigated. C-steel coupons left for 24 hours in the tested solutions containing and free of 10^−3^ M of I, II, and III surfactants and were taken to surface examination.

### 2.5 Computational details

The structure of the three synthesized surfactants were optimized using density functional theory (DFT). The B3LYP functional^54^ and 3-21g* basis set is used for optimization in gas and aqueous media. All calculations were done by G09 code.^55^ For the aqueous medium, the CPCM model was used.^56^

The quantum parameters such as the highest occupied molecular orbital (HOMO), the lowest unoccupied molecular orbital (LUMO), energy gap (Δ*E*). The global hardness (*η*), softness (*σ*), and the fraction of electron transferred (Δ*N*) which calculated using the following equations:5
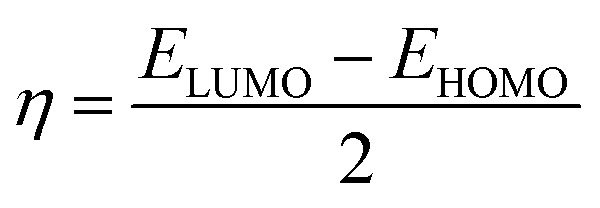
6
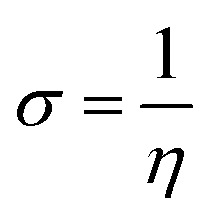
7
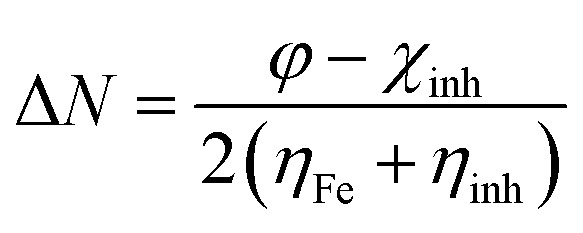
where *φ*, *χ*_inh_ are the work function (4.82 eV) and the electronegativity of inhibitors, respectively. *η*_Fe_, *η*_inh_ are the hardness of Fe (110) (0 eV) and hardness of inhibitors, respectively.

## 3. Results and discussion

### 3.1 FTIR spectrum

The structures of 5-{[6-(4-carboxybutoxy)-6-oxohexanoyl]oxy}pentanoic acid show absorption bands at 3320 cm^−1^ assigned to the (OH) group, 2910 and 2840 cm^−1^ ascribed to CH aliphatic, 1728 cm^−1^ assigned to C=O of the ester group, 1468 cm^−1^ C–H bond of CH_2_ group, and 1160 cm^−1^ ascribed to stretching of the C–O group (ESI† S1).

The structures of 1,6-bis(5-chloro-5-oxopentyl)hexanedioate show absorption bands at 2925 cm^−1^ ascribed to CH aliphatic, 1723 cm^−1^ assigned to C=O of the ester group, 1455 cm^−1^ C–H bond of CH_2_ group, and 1155 cm^−1^ ascribed to stretching of C–O group.

The structures of 1,6-bis({4-[bis(2-hydroxyethyl)carbamoyl]butyl})hexanedioate show absorption bands at 3360 cm^−1^ assigned to (OH) group, 2983 cm^−1^ ascribed to CH aliphatic, 1735 cm^−1^ assigned to C=O of the ester group, 1565 cm^−1^ assigned to stretching of C=O, 1470 cm^−1^ C–H bond of CH_2_ group, and 1180 cm^−1^ ascribed to stretching of C–O group (ESI† S2).

**Fig. 2:**
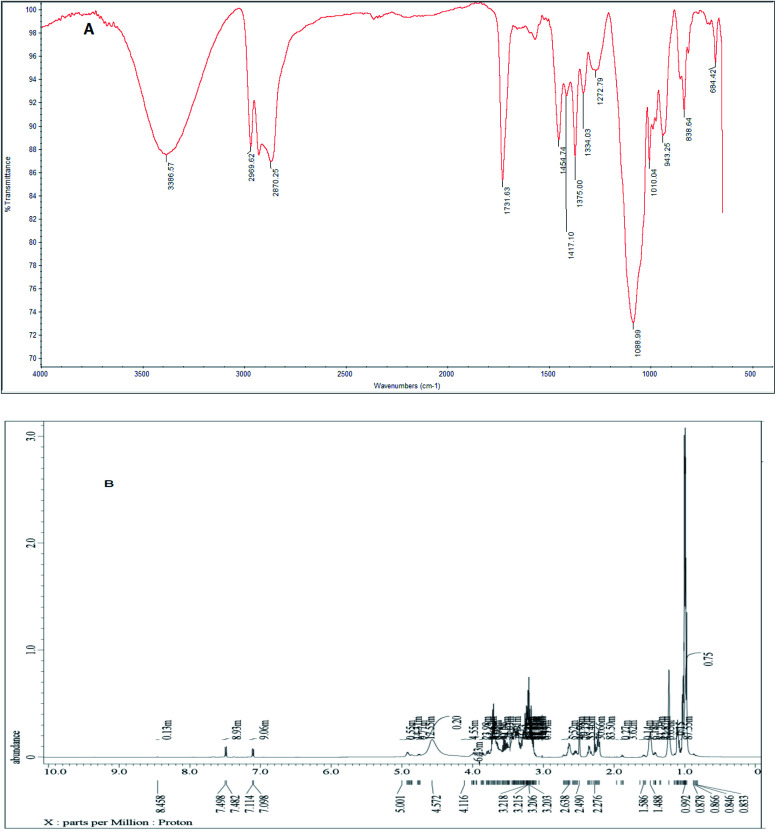
(A) FTIR spectra; (B) ^1^H-NMR of nonionic Gemini surfactants (I).

FTIR spectra were used to deduce the synthesized surfactants structures. The FTIR spectrum (Fig. 2A) of the synthesized nonionic Gemini surfactant (I) showed the following absorption bands at 3386 cm^−1^ were assigned to (OH) group, 2870 cm^−1^ were ascribed to CH aliphatic, 1731 cm^−1^ assigned to C=O of the ester group, 1454 cm^−1^ C–H bond of CH_2_ group and 1088 cm^−1^ were ascribed to C–O–C of ether group.

The FTIR spectrum of synthesized surfactant (II) displays absorption bands at 3340 cm^−1^ assigned to (OH) group, 2923 cm^−1^ ascribed to CH aliphatic, 1732 cm^−1^ assigned to C=O of the ester group, 1458 cm^−1^ C–H bond of CH_2_ group, and 1086 cm^−1^ ascribed to C–O–C of ether group (ESI† S3).

The FTIR spectrum of synthesized surfactant (III) displays absorption bands at 3464 cm^−1^ assigned to (OH) group, 2918 cm^−1^ ascribed to CH aliphatic, 1740 cm^−1^ assigned to C=O of the ester group, 1472 cm^−1^ C–H bond of CH_2_ group, and 1088 cm^−1^ ascribed to C–O–C of ether group (ESI† S4).

### 3.2 ^1^H-NMR spectra

^1^H-NMR (DMSO-d_6_) spectrum of the produced nonionic Gemini surfactants (I) (Fig. 2B) showed different peaks at *δ* = 0.878 ppm (t, 12H of –CH_3_); *δ* = 1.166 ppm (m, 16H of 2(CH̲_2_)_4_); *δ* = 1.488 ppm (m, 8H of N–CH̲_2_); *δ* = 2.638 ppm (t, 8H of CH̲_2_–O–C=O); *δ* = 3.203–3.218 ppm (m, 12H of repeated propylene oxide units); and *δ* = 7.114 ppm (broad S, 4H of OH).

The ^1^H-NMR (DMSO-d_6_) spectrum of the synthesized nonionic Gemini surfactants (II) showed different peaks at *δ* = 0.855 ppm (t, 12H of –CH_3_); *δ* = 1.314 ppm (m, 16H of 2(CH̲_2_)_4_); *δ* = 1.73 ppm (m, 8H of N–CH̲_2_); *δ* = 2.499 ppm (t, 8H of CH̲_2_–O–C=O); *δ* = 3.304–3.728 ppm (m, 12H of repeated propylene oxide units); and *δ* = 7.95 ppm (broad S, 4H of OH). (ESI† S5).

The ^1^H-NMR (DMSO-d_6_) spectrum of the produced nonionic Gemini surfactants (III) showed different peaks at *δ* = 0.853 ppm (t, 12H of –CH_3_); *δ* = 1.004 ppm (m, 16H of 2(CH̲_2_)_4_); *δ* = 1.244 ppm (m, 8H of N–CH̲_2_); *δ* = 2.498 ppm (t, 8H of CH̲_2_–O–C=O); *δ* = 3.133–3.575 ppm (m, 12H of repeated propylene oxide units); and *δ* = 7.944 ppm (broad S, 4H of OH). (ESI† S6).

### 3.3 Physical properties

**Fig. 3:**
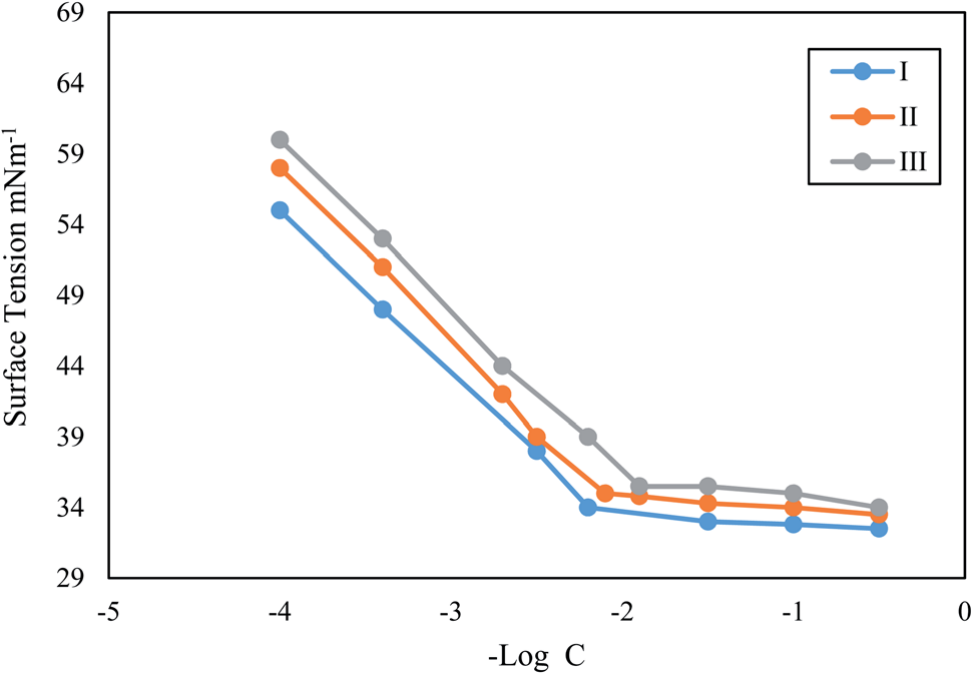
Dependence of surface tension on the concentration of the tested surfactants.

(CMC) values were derived from Fig. 3 and presented in Table 1. The lowest concentration at which surfactant monomers begin to aggregate and produce micelles is the (CMC) value. Values of surface pressure, maximum surface excess concentration, and minimum surface area occupied by one molecule of surfactant were calculated using eqn (8)–(10).^57,58^

8 *π*_CMC_ = *γ*_0_ − *γ*_CMC_

where (*γ*_0_) stands for pure water surface tension and (*π*_CMC_) is surfactant solution surface tension, at critical micelle concentration.9
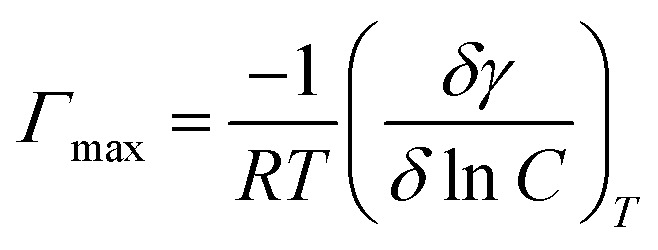
where *δγ* is surface pressure in mN m^−1^, and *C* is surfactant concentration. (*δγ*/*δ* ln *C*)_*T*_ is the slope of the plot of surface tension and concentration curves below CMC at a constant temperature.

10 *A*_min_ = 10^18^/*Γ*_max_*N*

**Table 1:** Surface properties of the synthesized Gemini surfactants

Comp.	Surface tension (mN m^−1^) 0.1 wt% at 25 °C	*γ*_CMC_ (mN m^−1^)	*π*_CMC_ (mN m^−1^)	CMC (mol L^−1^)	*Γ*_max_ × 10^−10^ (mol m^−2^)	*A*_min_ (nm^2^ mol^−1^)
I	32.0	33	39	0.110	8.698	19.08
II	33.5	35	37	0.122	8.298	20.20
III	34.0	35	37	0.149	7.663	21.66

*N* is the Avogadro's number, *π*_CMC_, *Γ*_max,_ and *A*_min_. Values of the nonionic Gemini surfactants are shown in Table 1. The effectiveness of surfactants decreases as surface tension rises at a critical micelle concentration. The nonionic Gemini surfactant III possesses the highest *A*_min_ value, whilst surfactant I possess the lowest.

### 3.4 Corrosion study

#### 3.4.1 Gravimetry measurements

Gravimetric experiments were carried out at various exposure time intervals with varying doses of the three produced surfactants inhibitors in a 1.0 M HCl solution. The information in Table 2 displays that the three surfactant compounds act as suitable inhibitors, wherein the inhibition performance will increase with the growing inhibitor's concentration. The inhibition performance for the three examined compounds increases withinside the order: I < II < III. Thus, this result could be related to the variety of propylene oxide units contained withinside the surfactant's molecule. As the propylene oxide units increase the molecular length increases covering a higher surface area and thus its inhibitive power increases.

**Table 2:** Corrosion parameters of C-steel in free and inhibited 1.0 M HCl solutions, as evaluated by weight loss. (CR) is the corrosion rate in g cm^−1^ h^−1^

Comp.	*t*, h	Free	1 × 10^−5^		5 × 10^−5^		1 × 10^−4^		5 × 10^−4^		1 × 10^−3^	
		CR	CR	*η*%	CR	*η*%	CR	*η*%	CR	*η*%	CR	*η*%
I	12	0.514	0.402	22	0.316	38	0.199	61	0.173	66	0.161	69
	24	0.614	0.401	35	0.294	52	0.198	68	0.173	72	0.159	74
	36	0.723	0.400	45	0.288	60	0.197	73	0.172	76	0.158	78
	48	0.822	0.387	53	0.283	65	0.197	76	0.171	79	0.157	81
	60	0.955	0.386	60	0.271	71	0.197	79	0.170	82	0.157	83
	72	1.062	0.385	64	0.271	74	0.197	81	0.162	85	0.156	85
	94	1.188	0.384	68	0.266	77	0.196	83	0.161	86	0.155	87
	106	1.301	0.383	71	0.255	80	0.196	85	0.160	88	0.154	88
	118	1.447	0.382	74	0.255	82	0.195	86	0.158	89	0.153	89
	130	1.607	0.381	76	0.249	84	0.194	88	0.158	90	0.152	90
II	12	0.514	0.436	15	0.359	30	0.233	55	0.182	64	0.147	71
	24	0.614	0.413	33	0.357	42	0.221	64	0.176	71	0.139	77
	36	0.723	0.400	45	0.352	51	0.221	69	0.171	76	0.130	81
	48	0.822	0.384	53	0.341	58	0.210	74	0.165	80	0.137	83
	60	0.955	0.378	60	0.335	65	0.198	79	0.152	84	0.136	86
	72	1.062	0.361	66	0.310	71	0.193	82	0.150	86	0.135	87
	94	1.188	0.333	72	0.277	77	0.182	85	0.143	88	0.135	89
	106	1.301	0.298	77	0.243	81	0.176	86	0.140	89	0.134	90
	118	1.447	0.269	81	0.223	84	0.160	89	0.135	91	0.133	91
	130	1.607	0.245	85	0.214	87	0.154	90	0.132	92	0.132	92
III	12	0.514	0.381	26	0.211	59	0.171	67	0.152	70	0.139	73
	24	0.614	0.380	38	0.210	66	0.170	72	0.151	75	0.138	78
	36	0.723	0.380	47	0.208	71	0.169	76	0.150	79	0.131	82
	48	0.822	0.377	54	0.207	75	0.167	79	0.147	82	0.129	84
	60	0.955	0.376	61	0.205	78	0.164	83	0.1468	85	0.129	87
	72	1.062	0.375	65	0.204	81	0.159	85	0.144	86	0.128	88
	94	1.188	0.374	71	0.203	83	0.158	87	0.142	88	0.127	90
	106	1.301	0.373	71	0.202	84	0.157	88	0.141	89	0.126	91
	118	1.447	0.372	74	0.201	86	0.156	89	0.137	90	0.125	92
	130	1.607	0.371	77	0.200	87	0.149	91	0.136	91	0.124	93

**Fig. 4:**
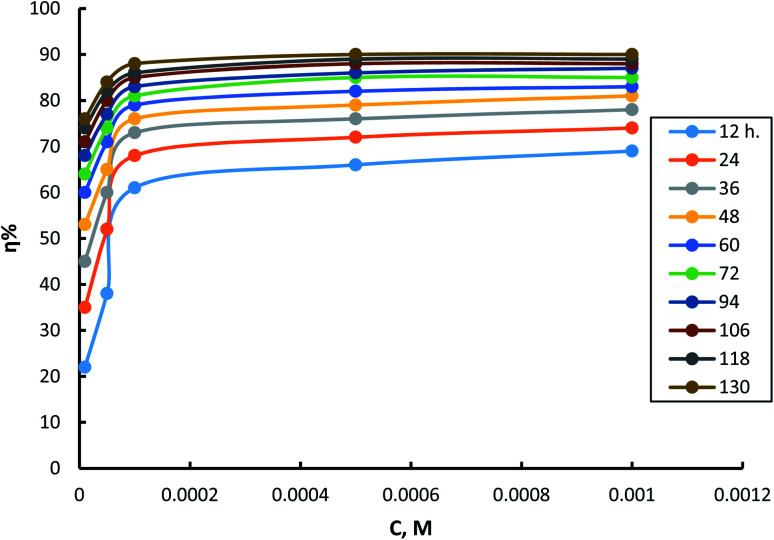
Dependence of inhibition efficiency on surfactant I concentration at different times of exposure.

Fig. 4 shows the dependence of inhibition power on surfactant (I) concentration. The same trends for surfactants II and III can be seen in (S7 and S8,† respectively). It is clear from this curve that inhibition power increases by increasing inhibitor concentrations. It is important to notice that the increment of inhibition power achieves its minimum rate as the inhibitor concentration reaches 10^−4^ M. This result suggests that just a very small concentration of adsorbed inhibitor molecules is needed to cover all the available sites on the metal surface. Thus, it could be concluded that every inhibitor molecule adsorbs at the surface of the steel in this type of manner that covers the highest possible surface area. The molecules of the surfactant are expected to adsorb horizontally at the steel surface in the currently tested compounds.

**Fig. 5:**
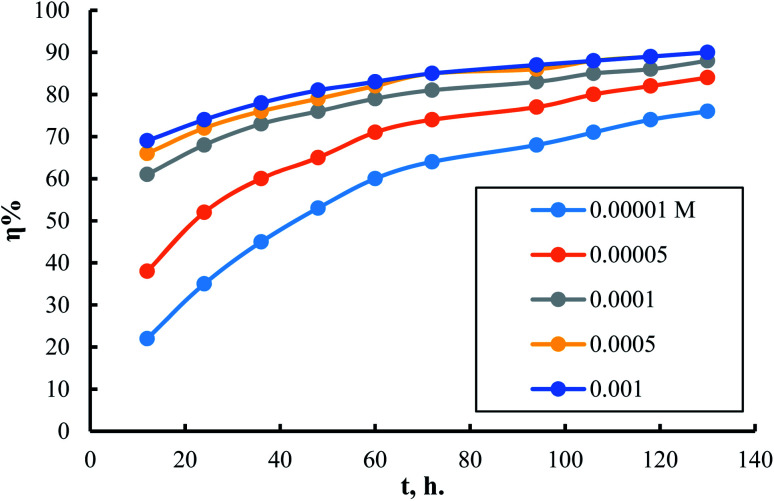
Dependence of inhibition efficiency on time of exposure for different concentrations of surfactant I.

The effect of exposure duration on the inhibitory power of different concentrations of surfactant (I) is shown in Fig. 5. The same figures for surfactants II & III are in (S9 and S10†). The figure shows that as the exposure time progresses on, inhibition efficiency improves. The increase in inhibition efficiency occurs at a rate that is higher in the cases of using low concentrations than using high concentrations. The increase in inhibition efficiency by increasing exposure time can be attributed, in all cases, to the presence of many surface sites available for adsorption at exposure start. Thus, over time, the number of surface sites occupied due to adsorption increases, and consequently the number of available surface sites decreases. It is obvious that with increasing inhibitor concentration at the start of exposure, the number of surface sites saturated with adsorbed molecules increases. So, the rate of inhibition efficiency increment is much lower in the case of high inhibitor concentrations than in lower ones.

#### 3.4.2 Potentiodynamic polarization

**Fig. 6:**
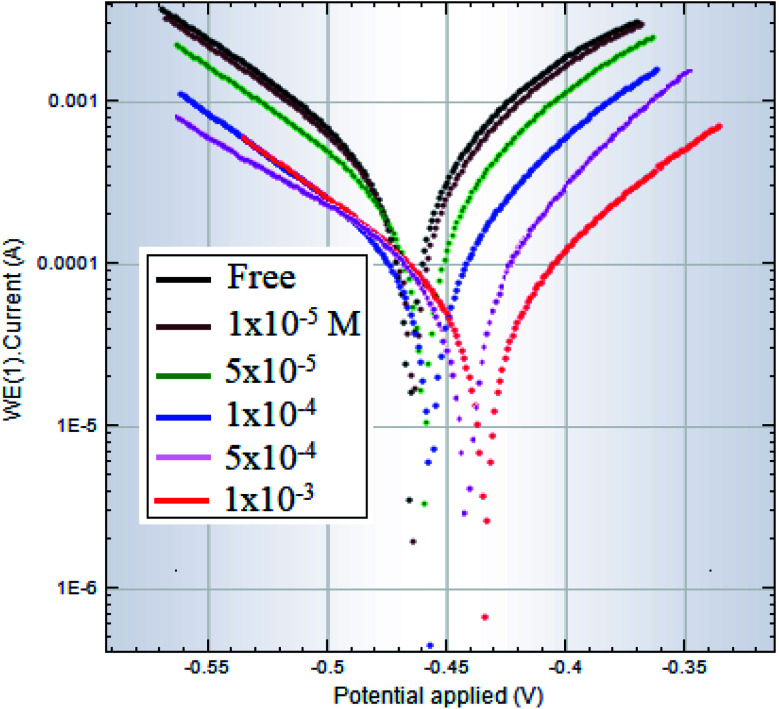
Polarization curves of C-steel in 1.0 M HCl solutions free and inhibited by different concentrations of surfactant I.

Fig. 6 displays polarization curves of a C-steel electrode in 1.0 M HCl free as well as inhibited by different surfactant (I) concentrations. Surfactants II and III have almost the same figures (S11 and S12†). When the surfactants are added, the polarization curves change towards less negative potentials and lower current densities, as shown in Fig. 6. This indicates that the surfactants investigated had an inhibiting effect on steel corrosion in an acidic medium.

Table 3 lists the data retrieved from polarization curves, containing corrosion current, corrosion potential, cathodic Tafel constant, anodic Tafel constant, in addition to inhibition efficiency. According to the results of Table 3, the studied surfactants behave as inhibitors of mixed type. The fact that the presence of the surfactant has almost no effect on the values of both Tafel constants leads to this conclusion. Besides, it indicates that the corrosion mechanism did not change by adding the inhibitor. Furthermore, the addition of the surfactant only mildly changes the corrosion potential toward less negative values. This shift of corrosion potential suggests also a mixed-type inhibition mechanism that act predominantly as anodic inhibitors.

**Table 3:** Polarization characteristics of C-steel corrosion in 1.0 M HCl solutions free of and containing different concentrations of the three surfactants

Comp.	Conc., (M) × 10^5^	−*E*_corr_, mV (SCE)	*β*_a_, mV per decade	−*β*_c_, mV per decade	*i*_corr_, μA cm^−2^	*η*%
	Free	467	90	85	306.36	—
I	1	459	88	66	88.09	71.25
	5	455	91	64	65.48	78.63
	10	452	96	68	59.91	80.44
	50	449	99	55	51.86	83.07
	100	452	98	61	49.58	83.81
II	1	463	89	75	87.65	71.45
	5	458	92	59	80.08	73.86
	10	453	86	73	43.41	85.83
	50	465	97	91	22.75	92.57
	100	461	91	85	21.84	92.87
III	1	460	92	78	38.31	87.50
	5	452	93	82	25.32	91.74
	10	449	97	95	24.77	91.91
	50	445	99	73	19.25	93.72
	100	460	94	81	18.56	93.94

Further checking of the Table 3, it is apparent that increasing the surfactant concentration increases the inhibitory efficiency. The inhibitive power increases in the following order for all three compounds tested: I < II < III. This sequence is the same as that the weight-loss technique revealed.

#### 3.4.3 Impedance spectroscopy

**Fig. 7:**
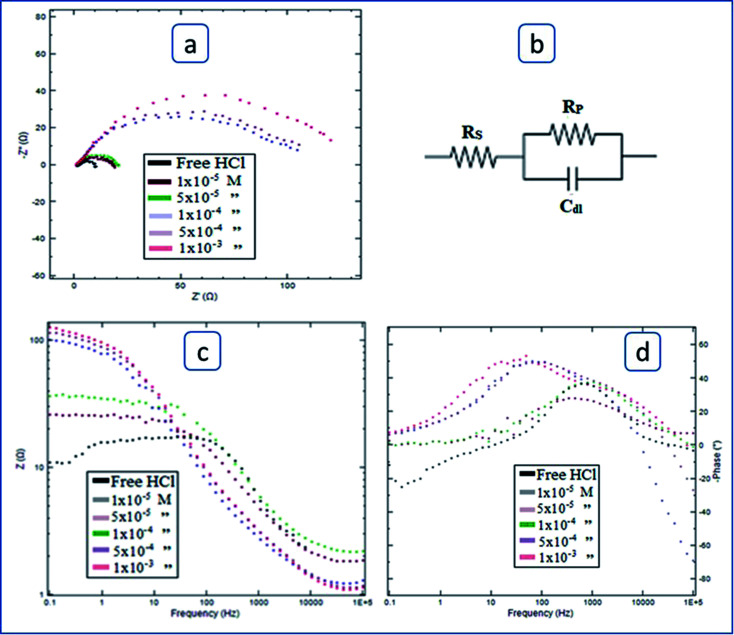
Carbon steel impedance spectra in 1.0 M HCl solutions without and with varying amounts of surfactant I. (a) Nyquist plot (b) equivalent circuit (c) Bode plot (d) frequency – phase angle relation.

Fig. 7 represents the carbon steel impedance spectra in 1.0 M HCl free as well as inhibited by different surfactant (I) concentrations, as well as the fitted equivalent circuit. Similar spectra corresponding to surfactants (II) and (III) are in the ESI† (S13 and S14, respectively). The Nyquist plot (Fig. 7a) shows depressed semicircles which have centers below the true axis. Increasing the surfactant concentration leads to an increase in the semicircle diameter.The equivalent circuit is shown in Fig. 7b. This result is evidence that the surfactant inhibits carbon steel acid corrosion. Moreover, this implies that acid steel corrosion is primarily a charge transfer process.^59^Fig. 7c represents the Bode plot which illustrates the dependence of impedance on the frequency. The figure clearly shows that carbon steel impedance increases by several orders of magnitude in inhibited acid solutions compared to free acid solutions. Impedance value increased as the surfactant's concentration is increased. Fig. 7d shows a peak of phase angle which increases and shifts toward low-frequency values with increasing surfactant concentration. It was stated that this shift in phase angle is attributed to the change of the metal interfacial structure owing to the development of surfactant molecules adsorbed layer.^60^

Table 4 contains the impedance parameters related to the corrosion process obtained from calculations based on the equivalent circuit that has been fitted, as shown in Fig. 7b. The data of the table show that the polarization resistance, and thus the inhibition power, increases as the concentration of surfactant is increased. It is worth noticing that the studied surfactants' inhibitory efficacy increases in the same sequence as that of weight loss and polarization techniques: I < II < III.

**Table 4:** Impedance characteristics of carbon steel in 1.0 M HCl solutions, devoid of and containing different concentrations of the tested compounds

Compound	Conc., (M) × 10^5^	*R*_S_ (Ω)	*R*_P_ (Ω)	*η*%	*N*
	Free	3.20	11.00	—	0.99885
Free	3.20	11.00	—	0.99885	
I	1	1.41	18.14	38.89	0.99509
	5	1.49	22.31	50.00	0.99396
	10	1.56	104.22	89.42	0.99301
	50	1.47	112.15	90.17	0.99198
	100	1.39	122.32	90.98	0.99474
II	1	1.74	23.88	35.93	0.99509
	5	2.01	34.46	68.07	0.9946
	10	1.25	105.80	89.60	0.99429
	50	1.69	118.48	90.71	0.99454
	100	1.67	128.53	91.44	0.99485
III	1	1.68	19.31	43.03	0.9942
	5	1.39	96.19	88.56	0.99296
	10	1.66	106.96	89.71	0.99466
	50	1.94	121.55	90.95	0.99413
	100	1.96	138.35	92.05	0.99434

### 3.5 Adsorption behavior

The adsorption of inhibitor molecules on the metal surface is the first stage in the inhibition process. At the metal/corrosive media interface, the inhibitor molecules form an isolating film. Adsorbed molecules maybe still with their chemical identity or combine with the dissolved metal cations, at the interface, forming a new chemical compound. The former case is called physical adsorption while the latter is chemical adsorption. The adsorption isotherm could be used to accurately describe the adsorption process's characteristics. A variety of adsorption isotherms were examined in this study using the results of weight loss studies. The Langmuir isotherm model is revealed to be the most suitable for the current data. As per the Langmuir isotherm model, the surface energy is influenced by the portion of the surface covered by inhibitor molecules (*θ*). Moreover, it assumes that there are no interactions between the molecules that have been adsorbed. The following equation describes the isotherm:11
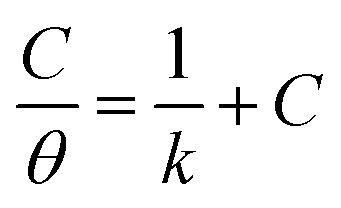


**Fig. 8:**
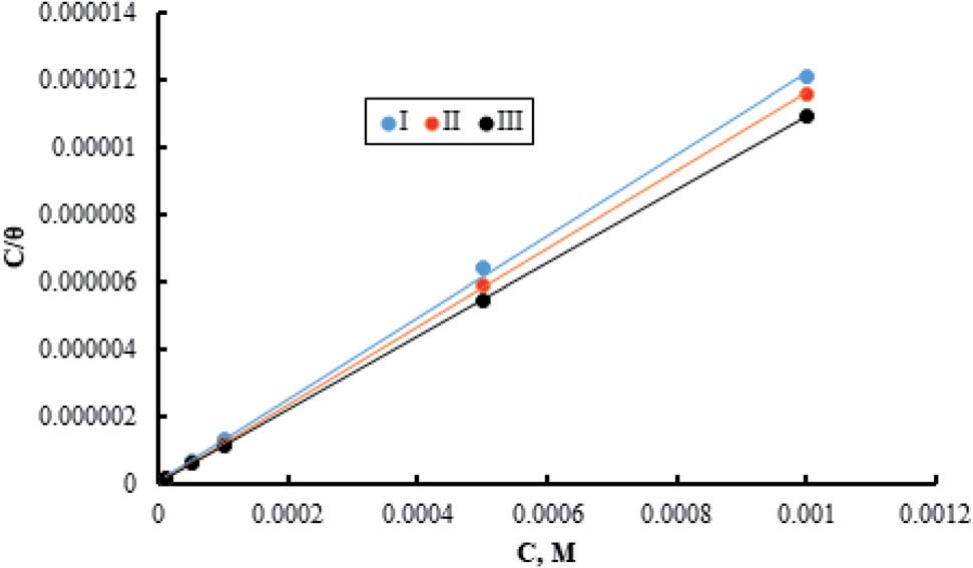
Langmuir adsorption isotherm for the three surfactants.

*C* denotes molar surfactant concentration, *θ* represents surface covering fraction while *k* is adsorption constant which is identified as:12
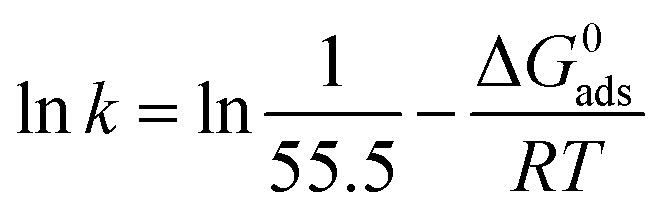
Δ*G*^0^_ads_denotes standard adsorption free energy while the numerical value 55.5 refers to water molar concentration. Fig. 8 shows a relation between the surfactant's molar concentration *C* and *C*/*θ*. The Langmuir isotherm of adsorption is confirmed by straight lines with unit value slopes. The calculated values of the standard adsorption free energies for the three surfactants are outlined in the Table 5.

**Table 5:** Standard free energy of adsorption

Surfactant	−Δ*G*^0^_ads_, kJ mol^−1^
I	49.90
II	51.61
III	53.89

Data in the Table 5 shows negative standard free energy values for all the tested surfactants. This indicates that the surfactant molecule's adsorption at the steel surface is a spontaneous process. Moreover, their values decline in the sequence: I > II > III, reflecting the tendency for adsorption process which in turn reflects the inhibitive capability. Therefore, this sequence is consistent with that obtained for inhibition efficiency.

### 3.6 Atomic force microscopy

Atomic force microscopy is a research instrument that provides information on the topography of a surface. In corrosion research, using such a methodology is advantageous because it gives a lot of data about the surface roughness of the metal under investigation. 2-D and 3-D photos of C-steel surface after exposition to 1.0 M HCl uninhibited solutions and inhibited by 10^−3^ M of each of surfactants examined shown in Table 6. The original-sized photos, as well as the associated comprehensive surface data, are available in (S15–S22†). Visual examination of the images reveals clearly that any surfactant presence significantly reduces the roughness of the carbon steel surface. Roughness diminishes in the following manner, depending on the sort of additives:

**Table 6:** 2-D and 3-D images of the carbon steel surface after exposure to 1.0 M HCl free of and containing 10^−3^M of the tested surfactants

	2D	3D
Free acid	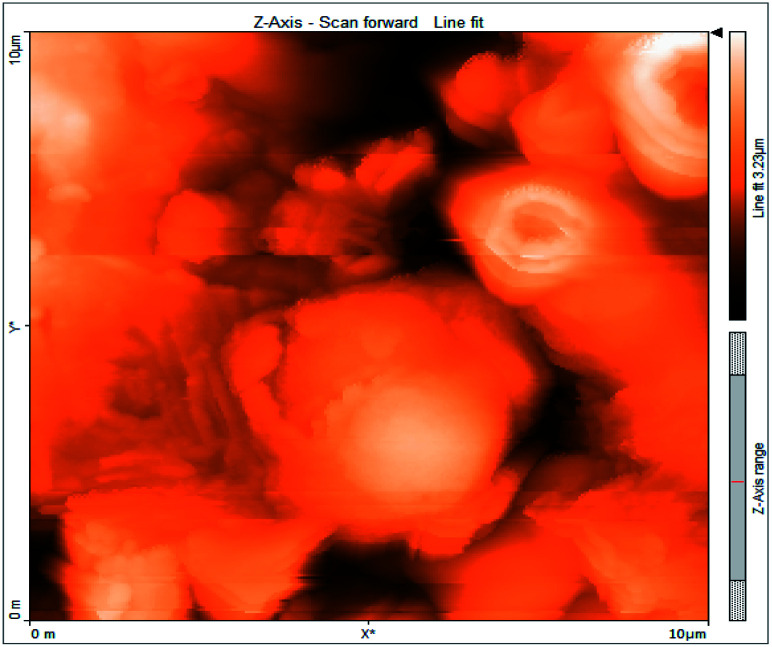	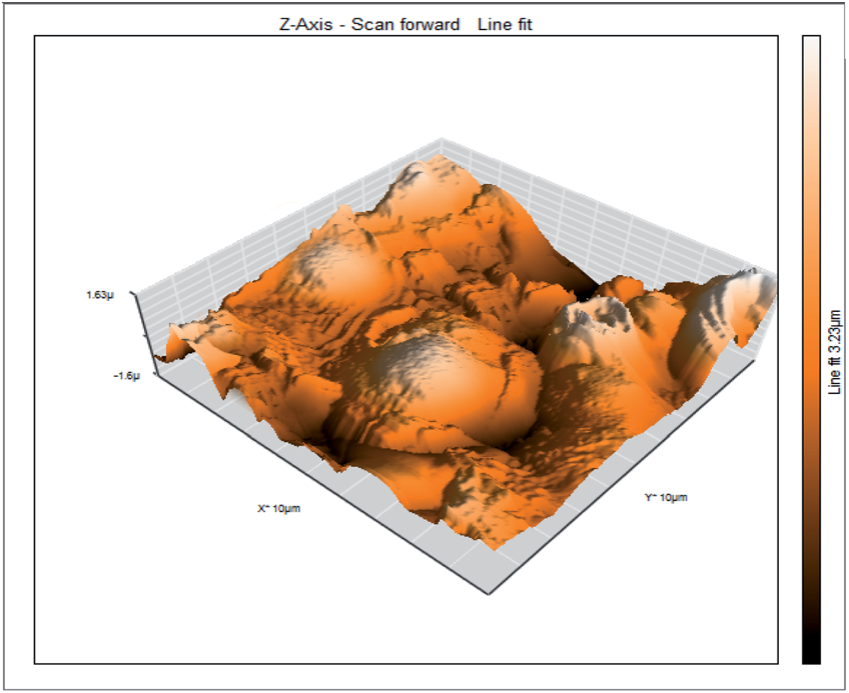
I	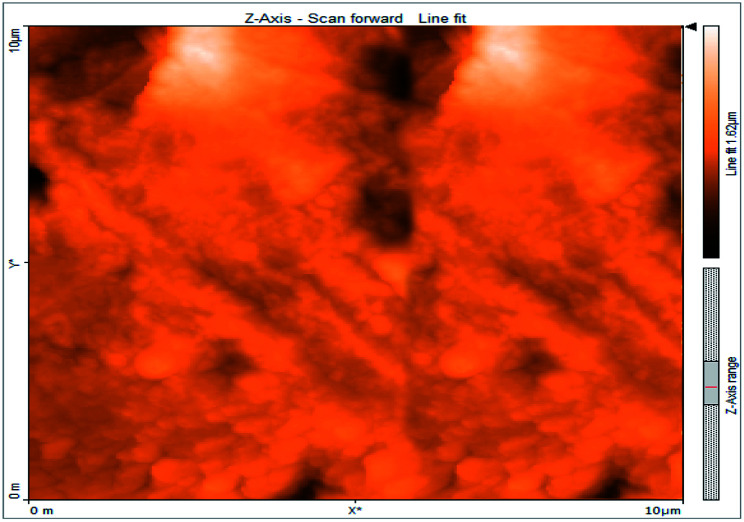	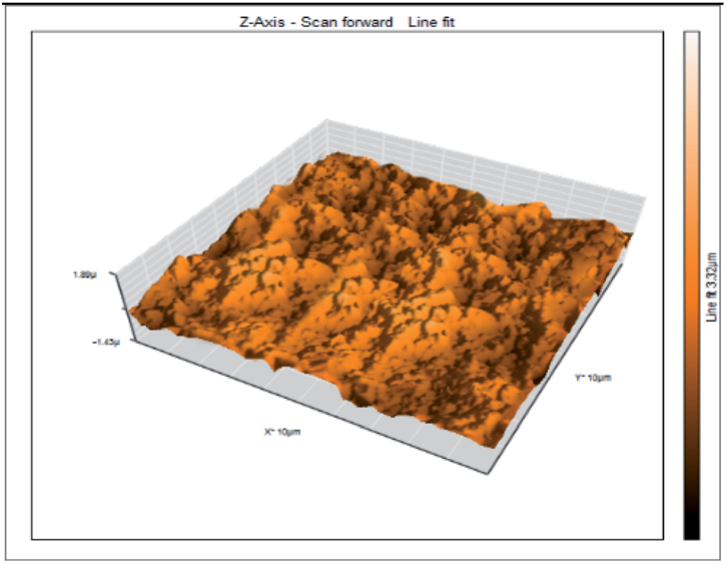
II	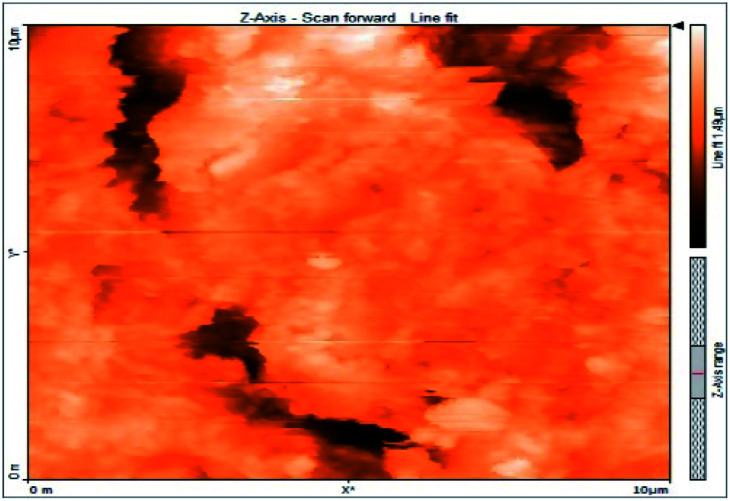	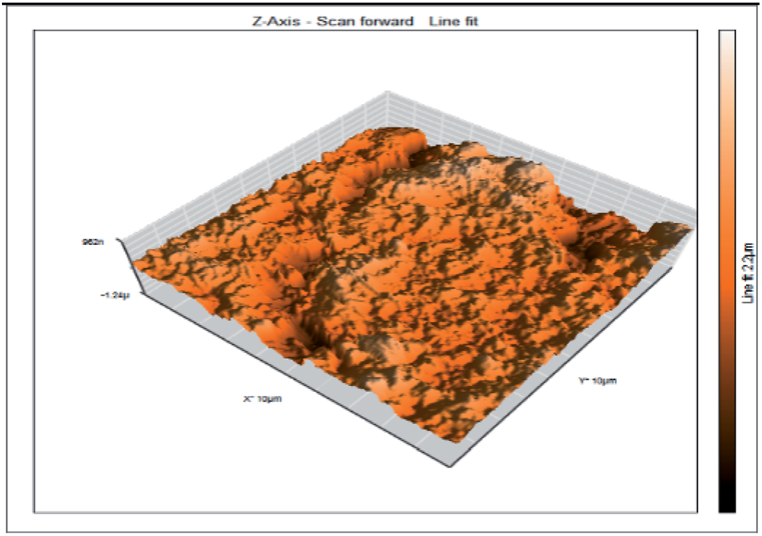
III	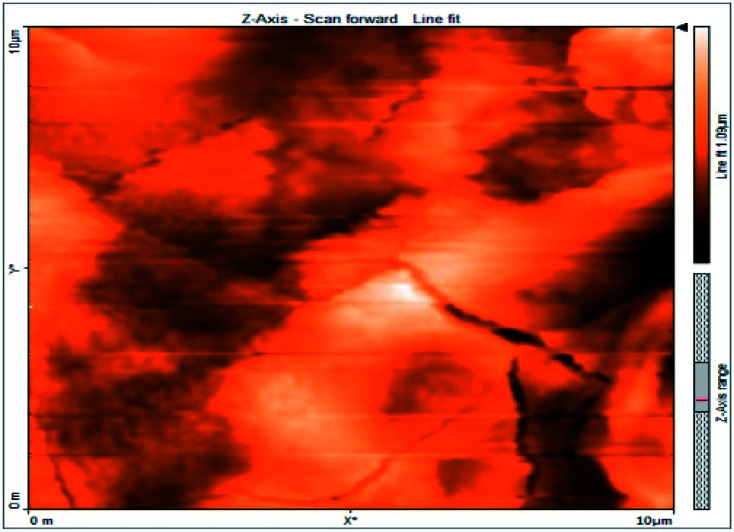	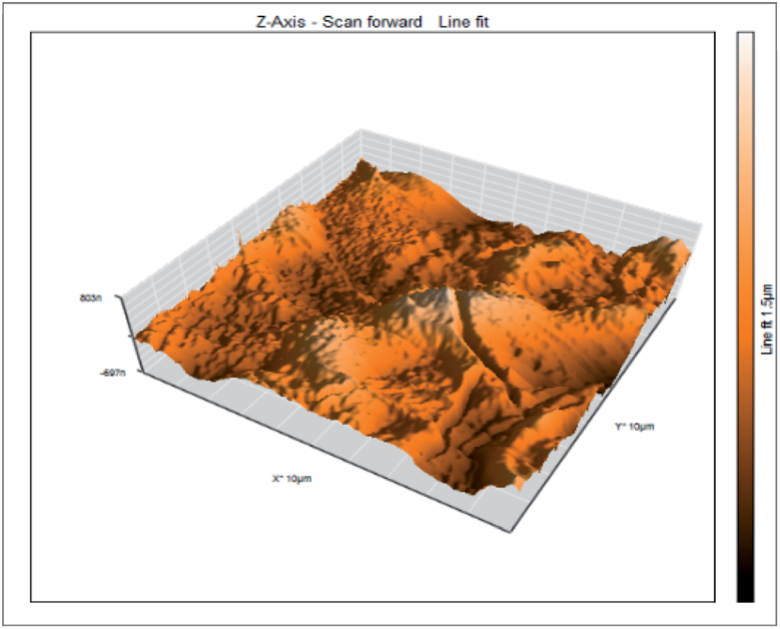

Free acid > free acid + I > free acid + II > free acid + III.

Table 7 shows the roughness of C-steel samples after one day of exposition to 1.0 M HCl solutions containing 1 × 10^−3^ M from every one of the tested surfactants at 25 °C. Roughness average (*S*_a_), peak height (*S*_p_), and valley depth (*S*_v_) are all displayed in the table. Table 7 shows that as progress from a free acid solution to an inhibited acid solution, all the measured values decline according to the following sequence:

**Table 7:** AFM roughness data of carbon steel surface after one-day exposure to 1.0 M HCl solutions free of and contains 1 × 10^−3^ M of surfactants (I–III), at 25 °C

	Free acid	I	II	III
Area, pm^2^	100.8	100.8	100.8	100.8
*S*_a_	978.76 nm	180.67 nm	164.18 nm	133.22 nm
*S*_p_	2710.1 nm	1145.1 nm	1332.2 nm	704.85 nm
*S*_v_	−5.0681 μm	−1090.6 nm	−560.59 nm	−565.41 nm

Free acid > free acid + I > free acid + II > free acid + III.

The addition of any of the three tested surfactants in the corrosive medium results in a smoother carbon steel surface. The surfactant molecules adsorbed on the carbon steel surface are responsible for this result. As a result, the effectiveness of the surfactant increases in the same way as that achieved by previous techniques: III > II > I.

### 3.7 Quantum calculations

**Fig. 9:**
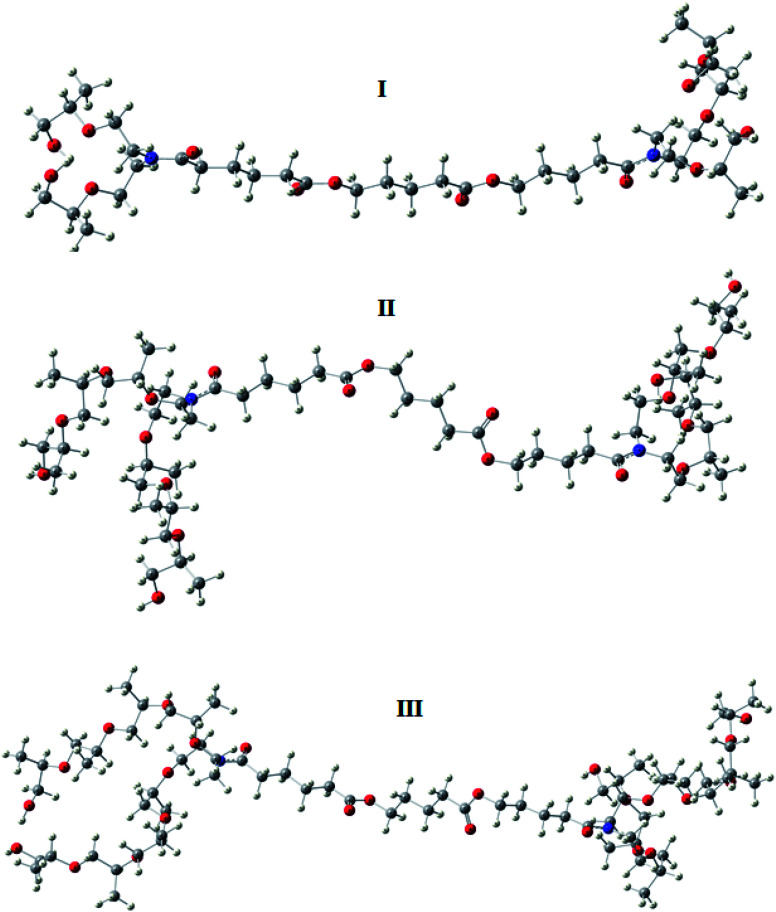
The optimized geometries of the surfactant compounds.

**Fig. 10:**
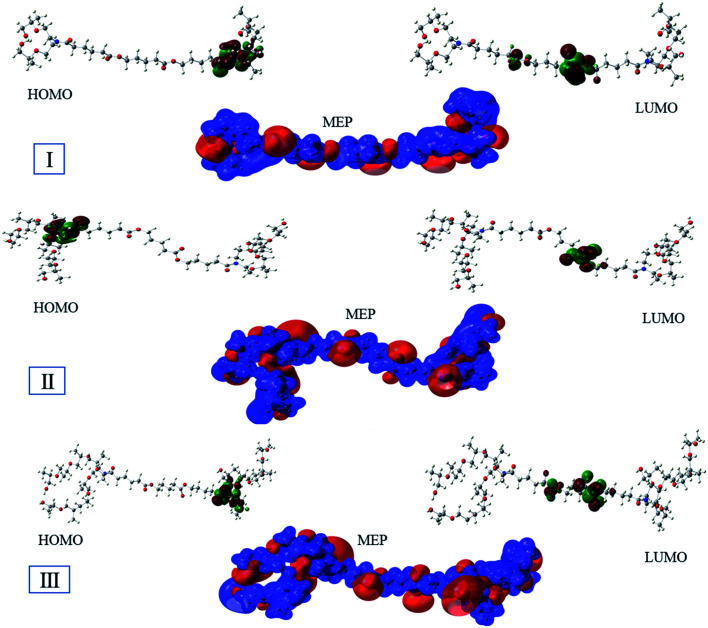
The Frontier orbitals and molecular electrostatic potentials of the surfactant compounds.

The optimized geometry of the three studied inhibitors were represented in Fig. 9. The Frontier molecular orbitals (HOMO and LUMO) and molecular electrostatic potentials (MEP) were seen in Fig. 10. The energies of HOMO and LUMO indicate electron-donating of the inhibitor to vacant 3d orbital of iron, and electron-accepting ability from the partially filled metal orbital, respectively.^61,62^ Therefore, a high value of HOMO energy and a low value of LUMO energy is associated with high inhibition efficiency. As shown in Table 8, inhibitor AE-15 in an aqueous medium has a higher value of HOMO and a lower LUMO value which gives the highest inhibition efficiency. The HOMO and LUMO orbitals of the three inhibitor molecules as seen in Fig. 10, it can be found that the HOMO electrons density is distributed on amid group in the three corrosion inhibitors, while the LUMO electron density is distributed over the ester group.

**Table 8:** Quantum parameters of the surfactant compounds

	Gas phase			Aqueous phase		
	I	II	III	I	II	III
*E*_HOMO_ (eV)	−5.89	−5.45	−5.32	−5.97	−5.88	−5.83
*E*_LUMO_ (eV)	0.46	0.51	0.59	0.31	0.27	0.26
Δ*E* (eV)	6.35	5.96	5.91	6.28	6.15	6.09
*η* (eV)	3.18	2.98	2.96	3.14	3.07	3.04
*σ* (eV^−1^)	0.31	0.34	0.34	0.32	0.33	0.33
Δ*N* (eV)	0.33	0.39	0.42	0.32	0.33	0.33

The energy gap Δ*E* is another descriptor that benefits from assessing the inhibitor's reactivity to the metal surface.^63^ The chemical reactivity is inversely proportional to the energy gap.^64^ The calculated values of Δ*E* under investigation are listed in Table 8, which can show the trends of Δ*E* for the three inhibitors as follow: I > II > III in both gas and aqueous media which agrees with the experimental results.

Also, global softness and hardness are important properties for measuring reactivity and molecular stability and can adsorb when adjacent to the metal surface. The inhibitors with a high value of softness and low value of hardness have a high capacity to adsorb on metal surfaces.^65,66^ As listed in Table 8, the III inhibitors possess a higher value of softness that have the highest inhibition efficiency.

The fraction of electrons transferred (Δ*N*), from Lukovits's study, which shows that the inhibition efficiency is due to the donation of electrons.^67^ If Δ*N* <3.6, the inhibition efficiency increases by increasing the electron donation of an inhibitor to the metal surface. As listed in Table 8, that III has the highest positive value of Δ*N* in the gas and aqueous phases which is compatible with the experimental result.

MEP is considered an important indicator that reflects the reactive sites of electrophilicity and nucleophilicity. The blue and red regions indicate the positive and negative electrostatic potentials, respectively. As shown in Fig. 10, where the negative electrostatic potential is concentrated on the oxygen and nitrogen atoms, and the positive electrostatic potential is located on the whole region of the inhibitor molecules.

**Fig. 11:**
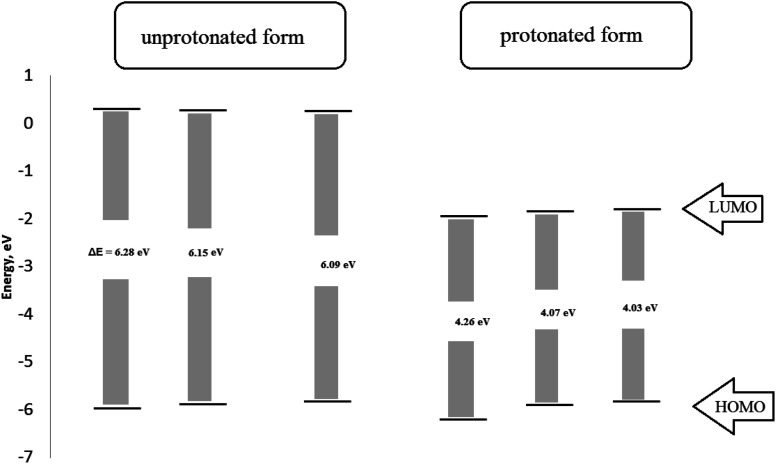
The Frontier orbital energies of protonated and unprotonated form for the studied surfactants.

The molecular structure containing heterogeneous atoms undergo protonation in acidic solutions. The nitrogen atoms of the amide group become a preferred site for protonation which have a higher negative charge derived by Milliken atomic charges. As shown in Fig. 11, the HOMO and LUMO of the unprotonated compared with protonated form. The HOMO values for the three investigated inhibitors decrease which suggests lowering affinity to donate electrons. LUMO values decrease indicating a higher tendency of accepting electrons. The bandgap in the protonated form has lower values than unprotonated suggesting the inhibitors become easily adsorbed on the metal surface in protonated form.

### 3.8 Inhibition mechanism

Results obtained from this study could be summarized in the following:

- The tested three Gemini surfactants showed high inhibition performance as for C-steel acid corrosion. With increasing concentration, the efficiency of inhibition improves up to a definite value after which it remains almost constant.

- A very small concentration of the surfactant is quite enough for reaching the most attainable value of efficiency.

- The efficiency of inhibition increases with increasing the exposure time. The dependence of efficiency of inhibition on exposure time is higher for low surfactants concentrations than higher ones.

- The efficiency of inhibition rises in the sequence: I < II < III illustrating how the molecule's length affects the process.

- The tested surfactants behave as mixed-type inhibitors.

- The calculated parameters obtained from DFT agree with the experimental results.

Based on the information acquired thus far, a conclusion has been reached about the corrosion inhibition mechanism. As the carbon steel is brought into contact with the inhibited acid solution, the surfactant molecules adsorb at anodic as well as cathodic locations on the steel surface. By blocking the transmission of charge and mass between steel and the corrosive medium, the adsorbed molecules form an insulating layer retarding both anodic and cathodic half-reactions. The surfactant's molecules adsorb horizontally covering high surface areas, so the maximum available sites were occupied just by a very small concentration of the inhibitor based on the number of surfactant molecules, the high surfactant concentrations achieve almost their highest inhibition effect at a relatively small exposure time. On the other hand, surfactants with lower concentrations still depend on exposure time to bring enough molecules from the bulk solution to the interface region.

Gemini surfactants with their unique molecular structure that has oxygen and nitrogen atoms having lone pairs of electrons dispersed throughout their skeletons. This configuration encourages the surfactant molecules to horizontal adsorption on the steel surface. The comparatively high *A*_min_ values (Table 1) additionally support this adsorption mode. In such an adsorption route, the inhibitor molecules can cover a high surface area of the steel through relatively low concentration.

## 4. Conclusion

Three novel Gemini surfactants based on adipic acid were synthesized with different numbers of propylene oxide units. Their structures were characterized by IR and NMR spectra. Experimentally, their physical properties were determined. Detailed experiments were conducted to determine their inhibitory effects on carbon steel acid corrosion. It was found that all of them can retard steel corrosion with high efficiencies. The inhibition process was found to be dependent on the surfactant concentration, exposure time, and molecular length. The theoretical calculation made to correlate their inhibition effect with their structures agreed with the experimental results and shed more light on the mechanism of their action.

## Author contributions

Mohamed Deef Allah was involved in the synthesis of the surfactants and made the measurements of their physical properties. Samar Abdelhamed and Mona A. El-Etre were involved in the corrosion measurements and wrote the discussion of the results as well as the inhibition mechanism part. Kamal A. Soliman was involved in the theoretical calculation and made the correlation between the structures and the inhibition efficiency. All the authors share in writing, review and editing the manuscript.

## Conflicts of interest

The authors declare no financial or commercial conflict of interest.

## Supplementary Material

RA-011-D1RA07449K-s001
