# Supplementary material for: The performance of three novel Gemini surfactants as inhibitors for acid steel corrosion: experimental and theoretical studies
Source: RSC Adv. 2021 Nov 22;11(59):37482–97. doi: 10.1039/d1ra07449k (PMC9043746; doi:10.1039/d1ra07449k)
Supplement: RA-011-D1RA07449K-s001 [file RA-011-D1RA07449K-s001.pdf]

Electronic Supplementary Material (ESI) for RSC Advances.
This journal is © The Royal Society of Chemistry 2021

The performance of three novel Gemini surfactants as inhibitors for acid steel corrosion: Experimental and theoretical studies

Mohamed Deef Allah[1*], Samar Abdelhamed[1], K.A. Soliman[3], Mona A. El-Etre[2]

[1]Basic Science Department, Faculty of Engineering, Shoubra, Benha University, Egypt

[2]Basic Science Department, Faculty of Engineering, Benha, Benha University, Egypt

[3]Chemistry department, Faculty of Science, Benha, Benha University, Egypt

[*]Corresponding author email: hamoaaa2002@yahoo.com

Supplementary materials

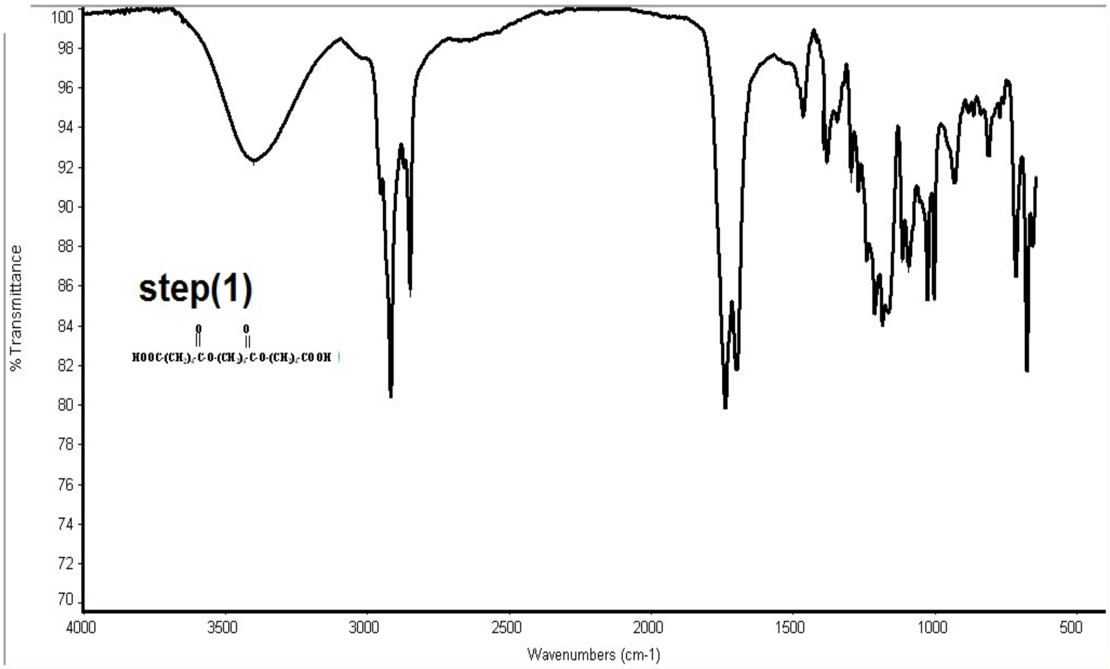

S1: FTIR of 5-{[6-(4-carboxybutoxy)-6-oxohexanoyl]oxy}pentanoic acid

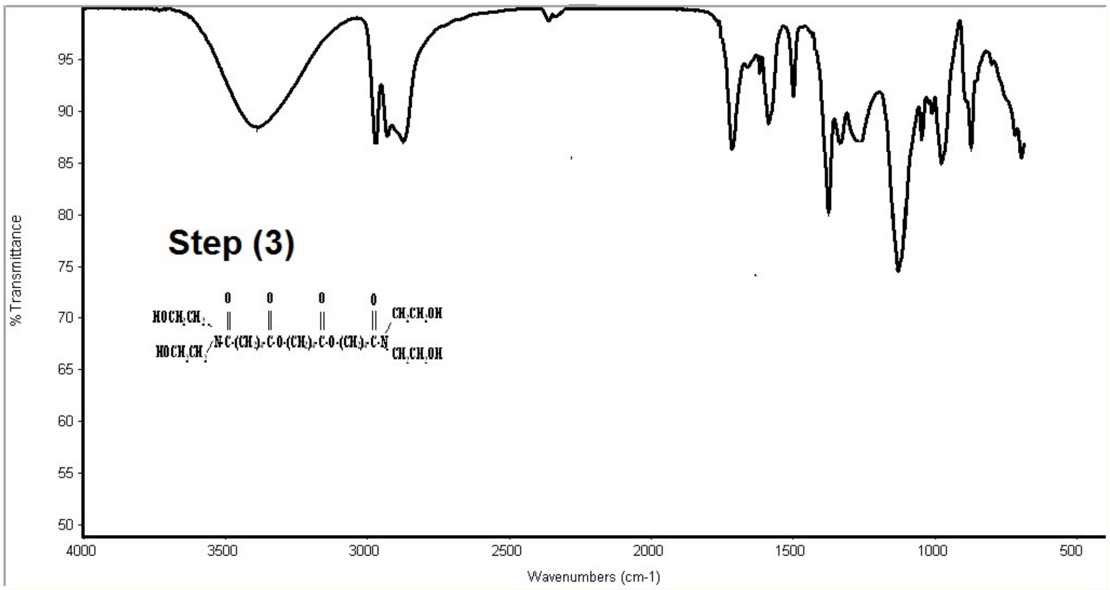

S2: FTIR 1,6-bis({4-[bis(2-hydroxyethyl)carbamoyl]butyl}) hexanedioate

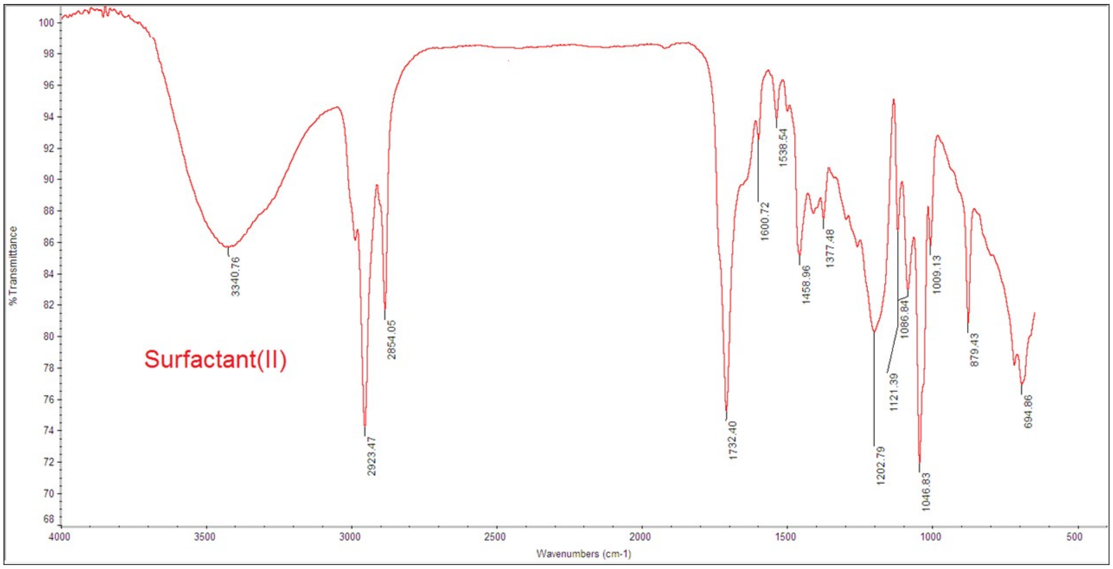

S3: FTIR of (II) nonionic Gemini surfactant.

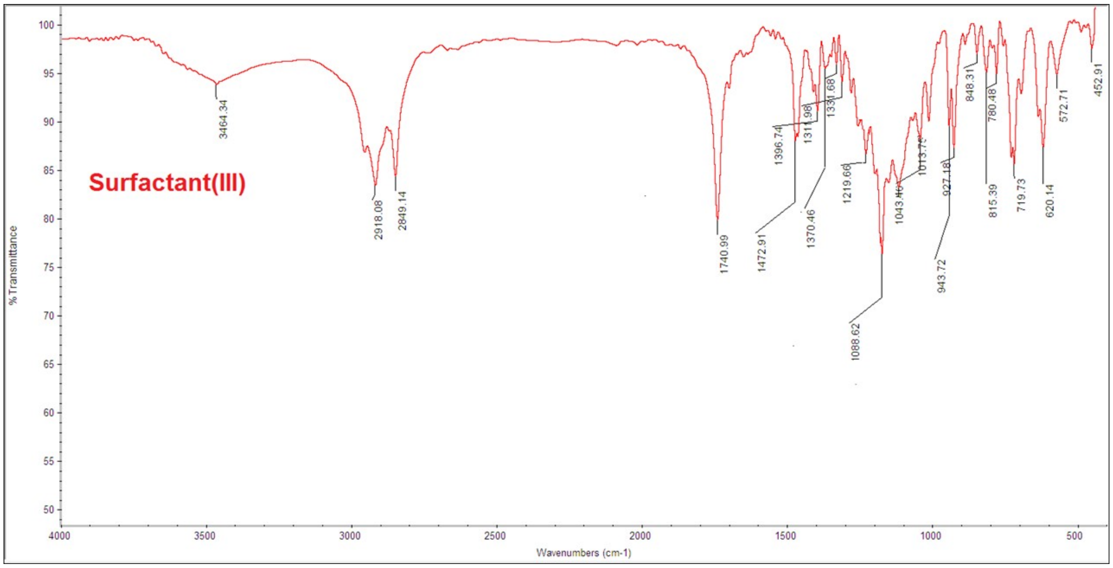

S4: FTIR of (III) nonionic Gemini surfactant.

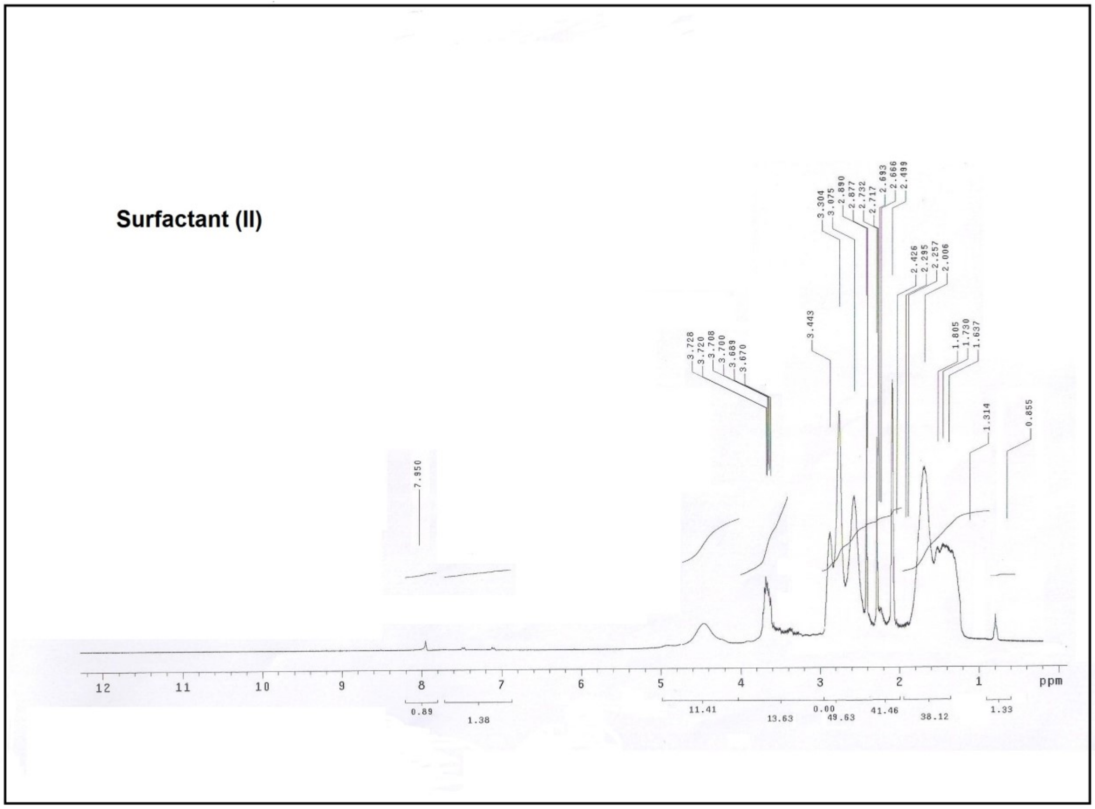

S5: ^{1}H-NMR of (II) nonionic Gemini surfactant

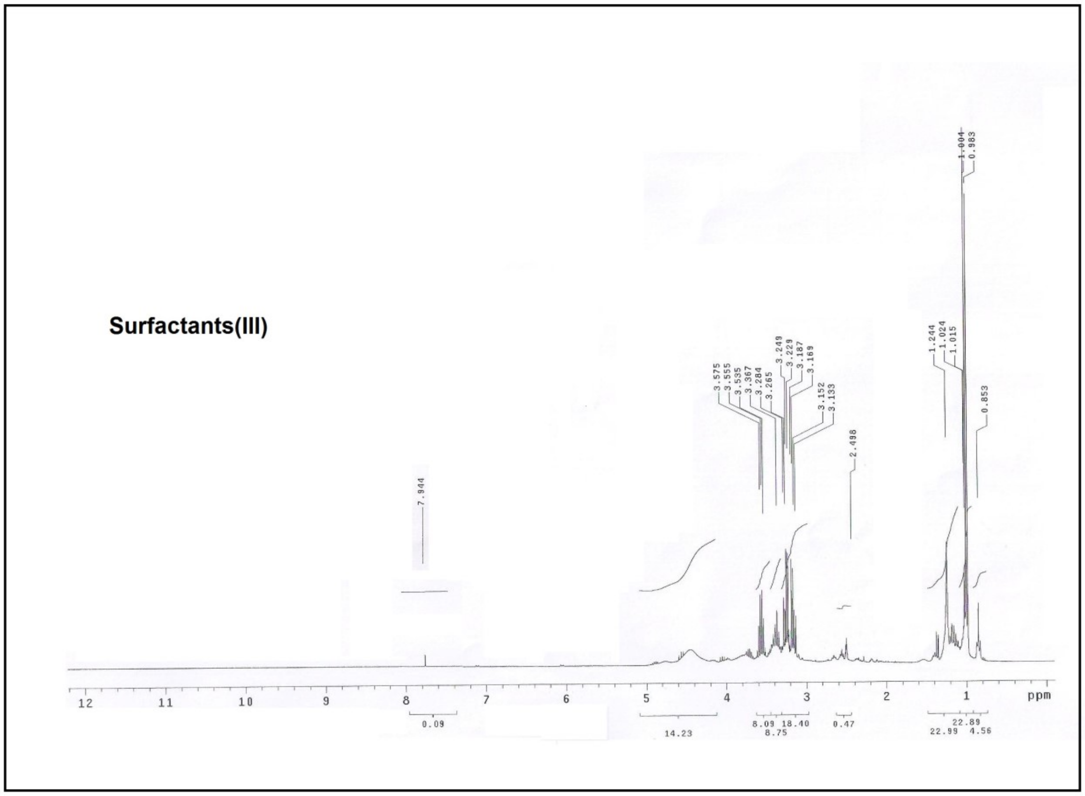

S6: ^{1}H-NMR of (III) nonionic Gemini surfactant.

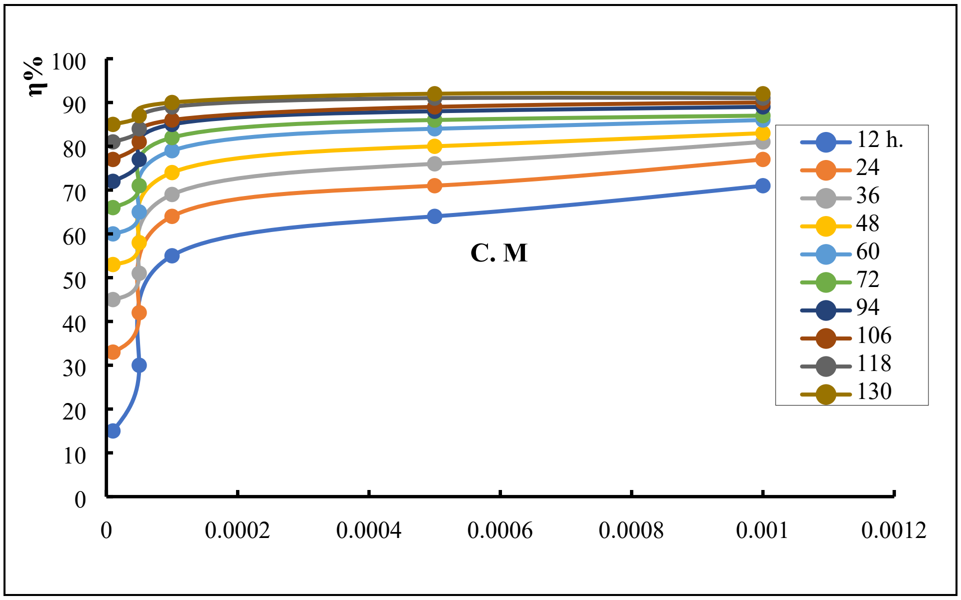

S7: Relationship between inhibition efficiency and compound II concentrations at different exposure times.

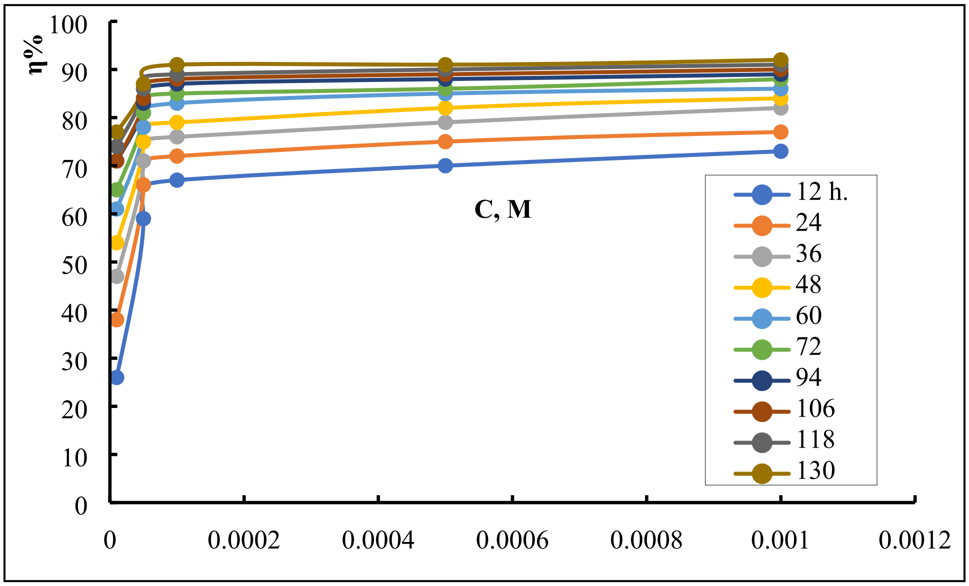

S8: Relationship between inhibition efficiency and compound III concentrations at different exposure times.

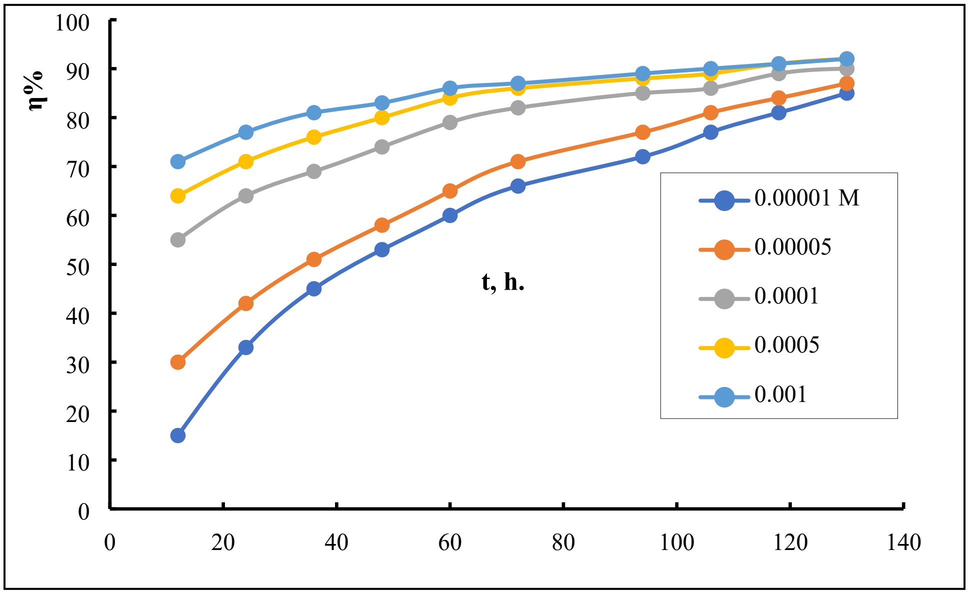

S9: Relationship between inhibition efficiency and exposure time for different concentrations compound II.

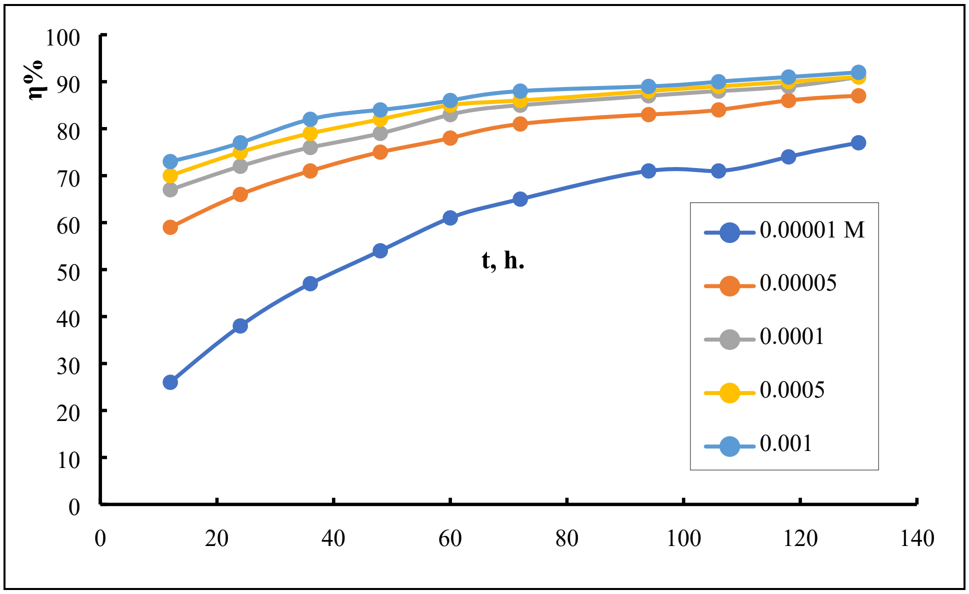

S10: Relationship between inhibition efficiency and exposure time for different concentrations compound III.

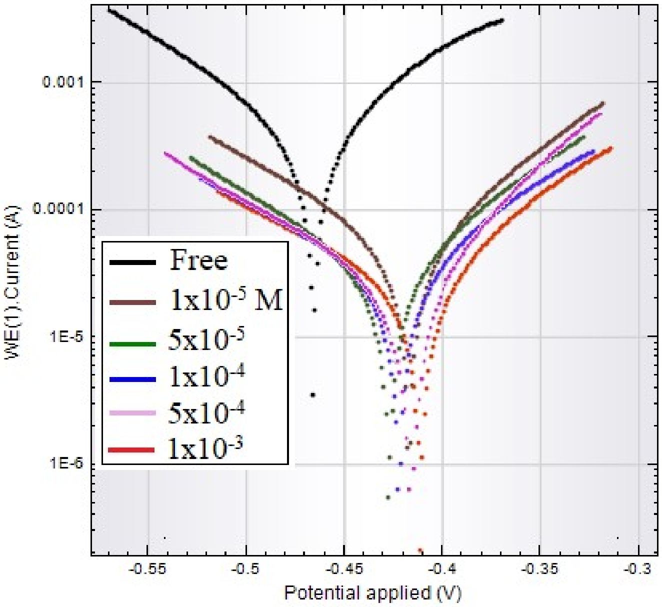

S11: polarization curves of C-steel in 1.0M HCl solutions free and inhibited by different concentrations of compound II.

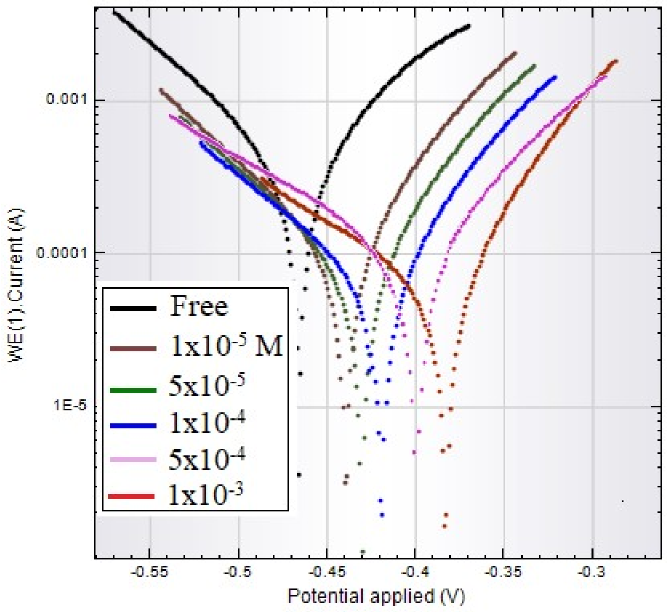

S12: polarization curves of C-steel in 1.0M HCl solutions free and inhibited by different concentrations of compound III.

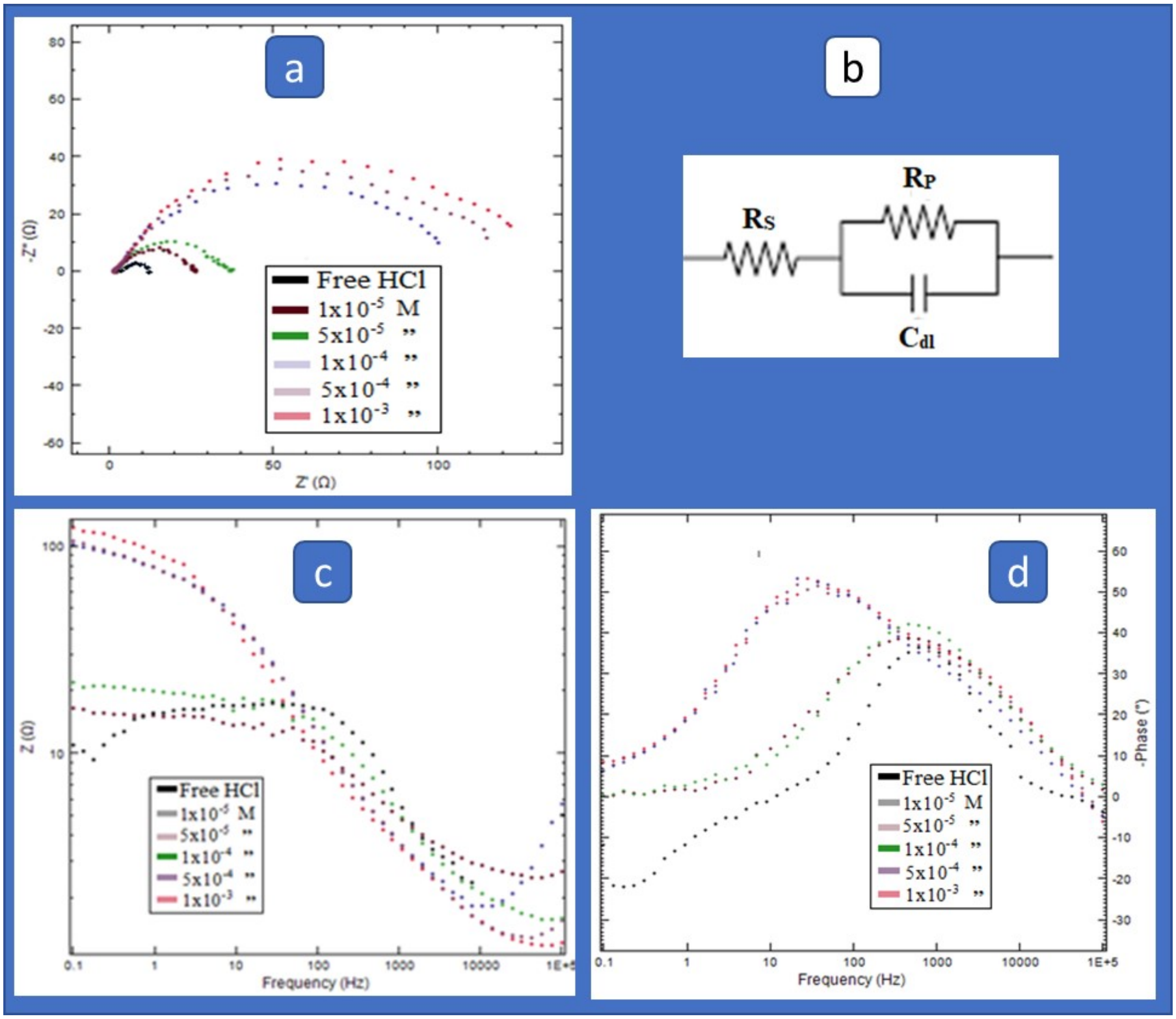

S13: Impedance spectra of carbon steel in 1.0 M HCl solutions avoid and containing different concentrations of compound II.

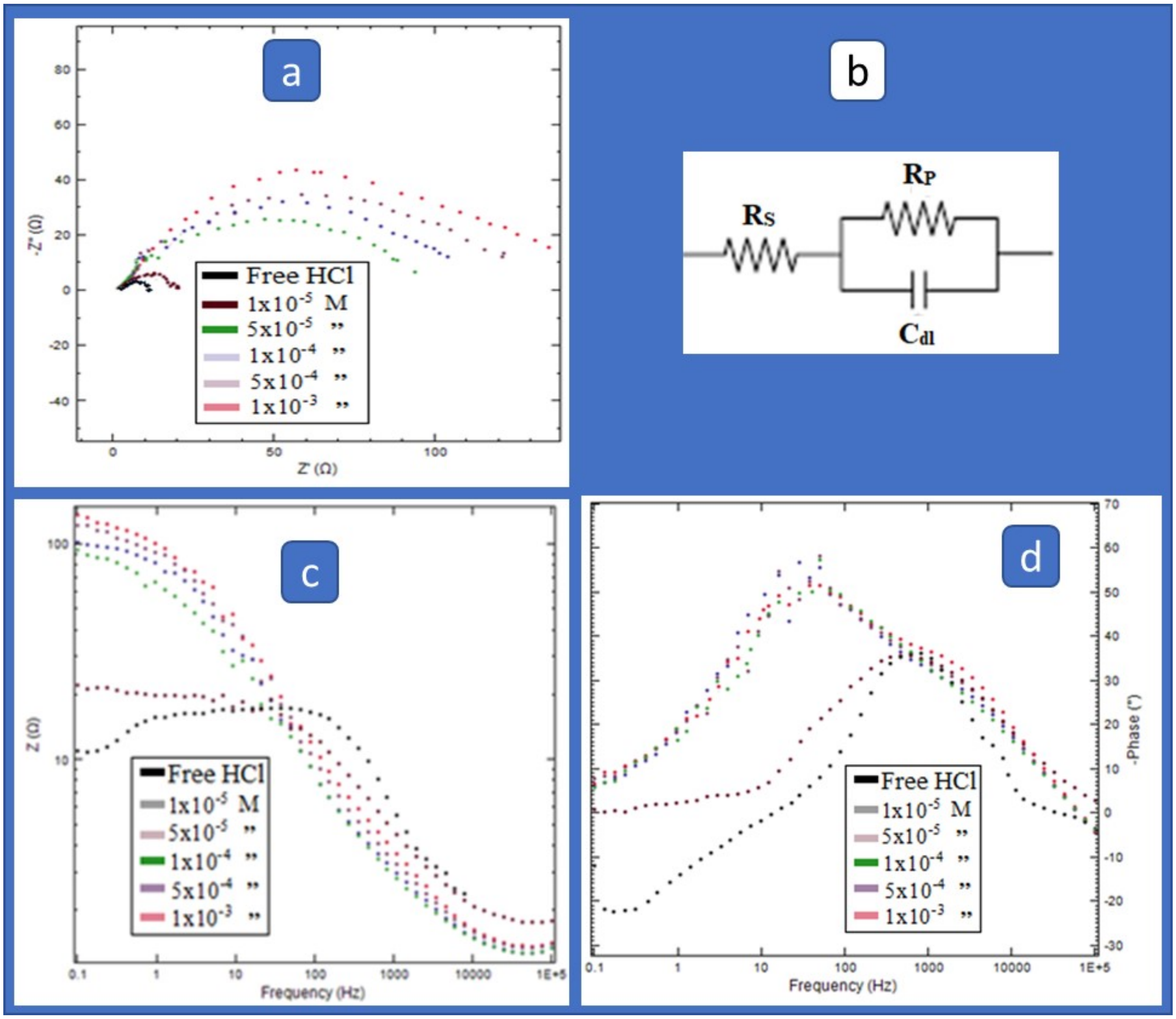

S14: Impedance spectra of carbon steel in 1.0 M HCl solutions avoid and containing different concentrations of compound III.

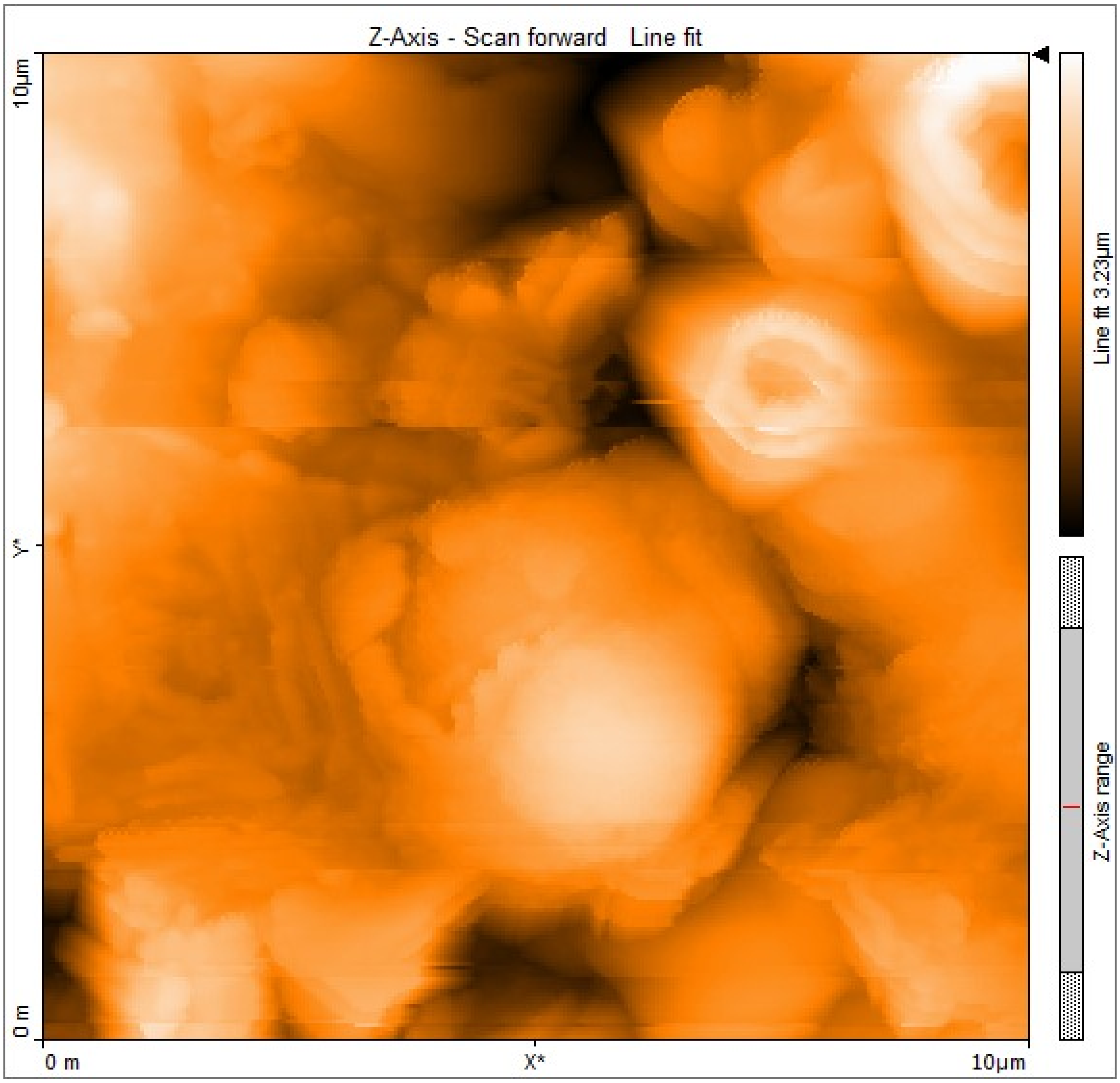

S15: 2-d image (1.0 M HCl)

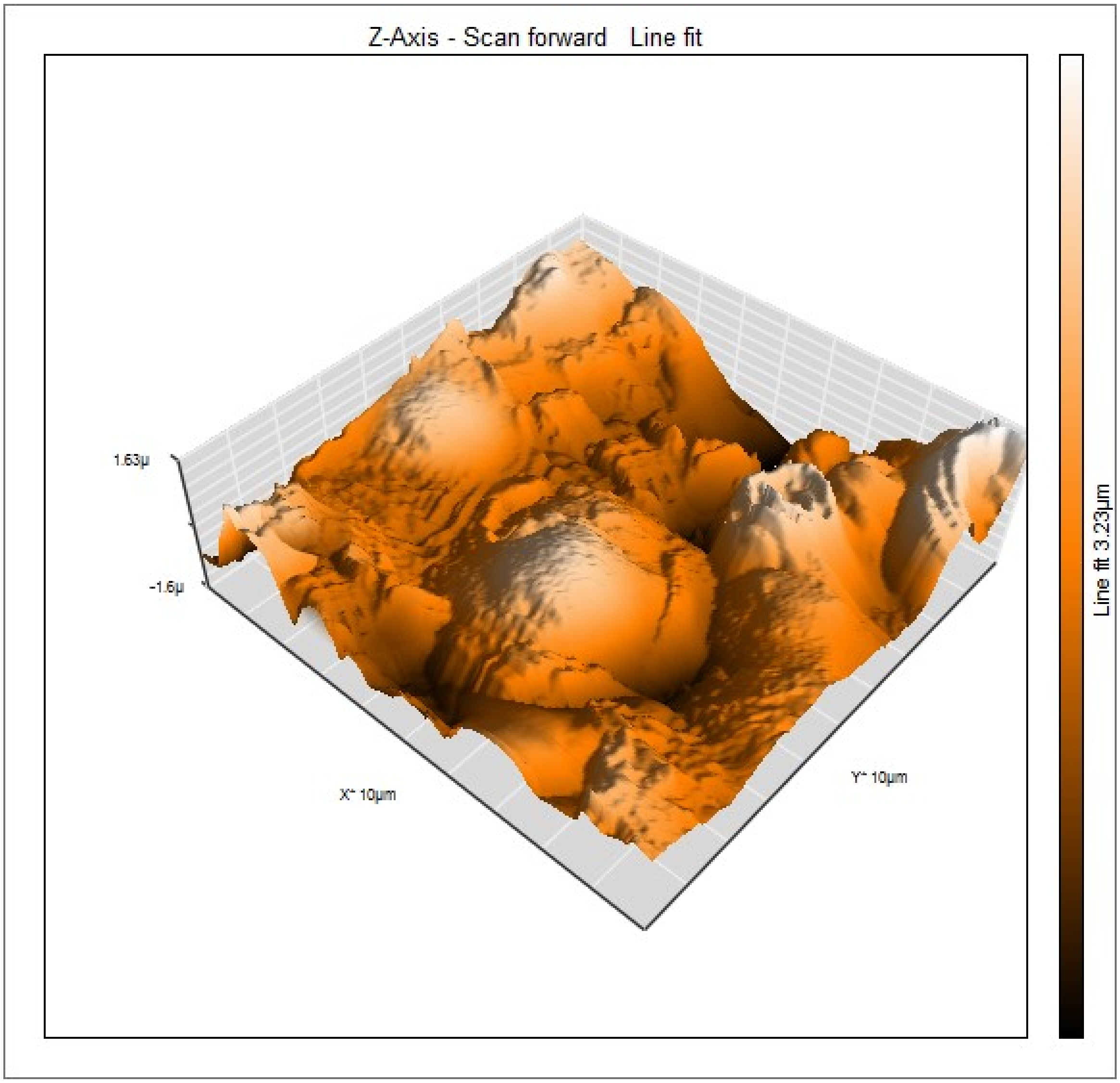

S16: 3-d image (1.0 M HCl)

Tool result

Name	Value
Area	100.8pm^2
Sa	978.76nm
Sq	1234.9nm
Sy	7.7781µm
Sp	2710.1nm
Sv	-5.0681µm
Sm	11.064pm

Store

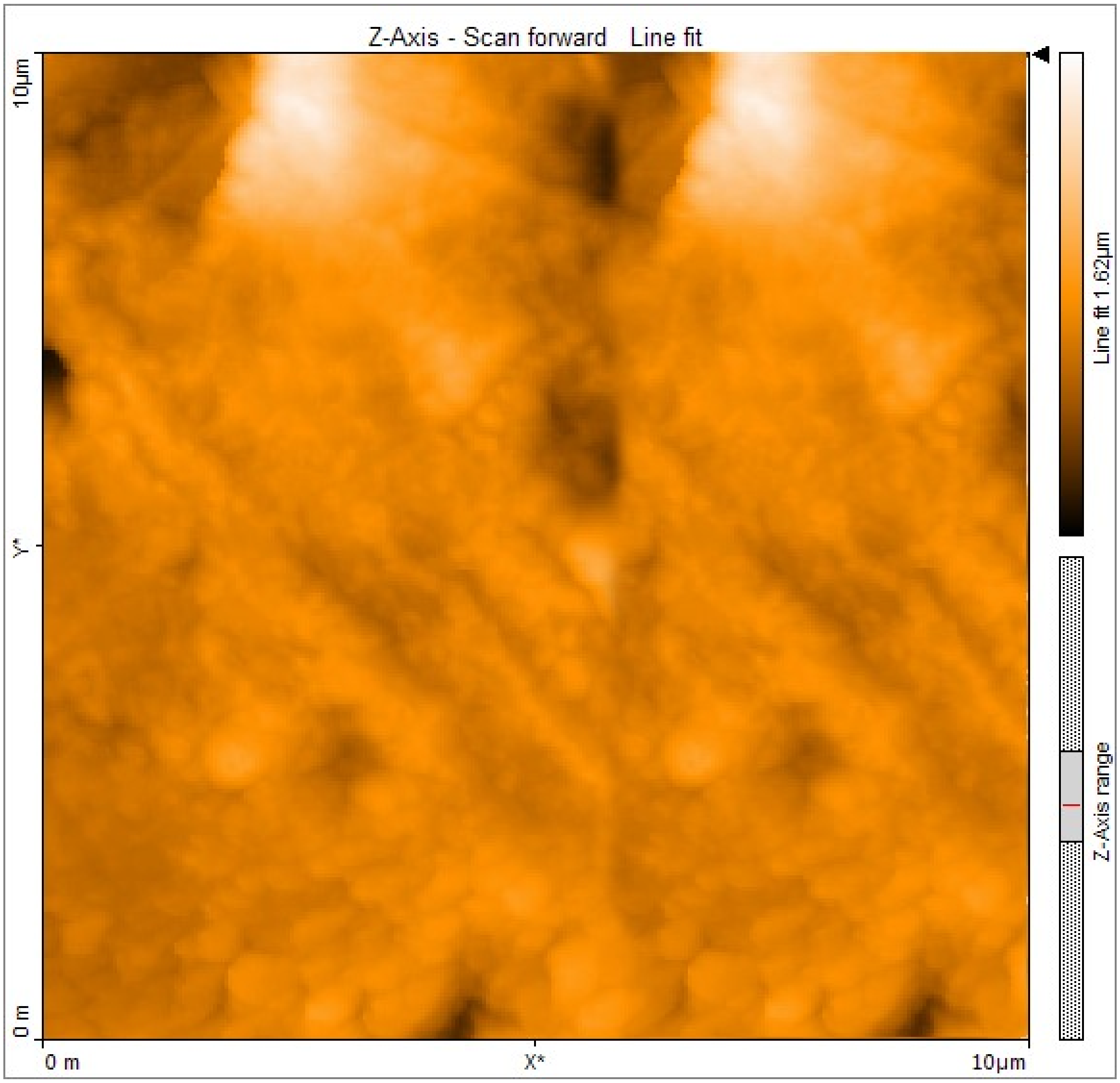

S17: 2-d image (1.0 M HCl + surfactant I)

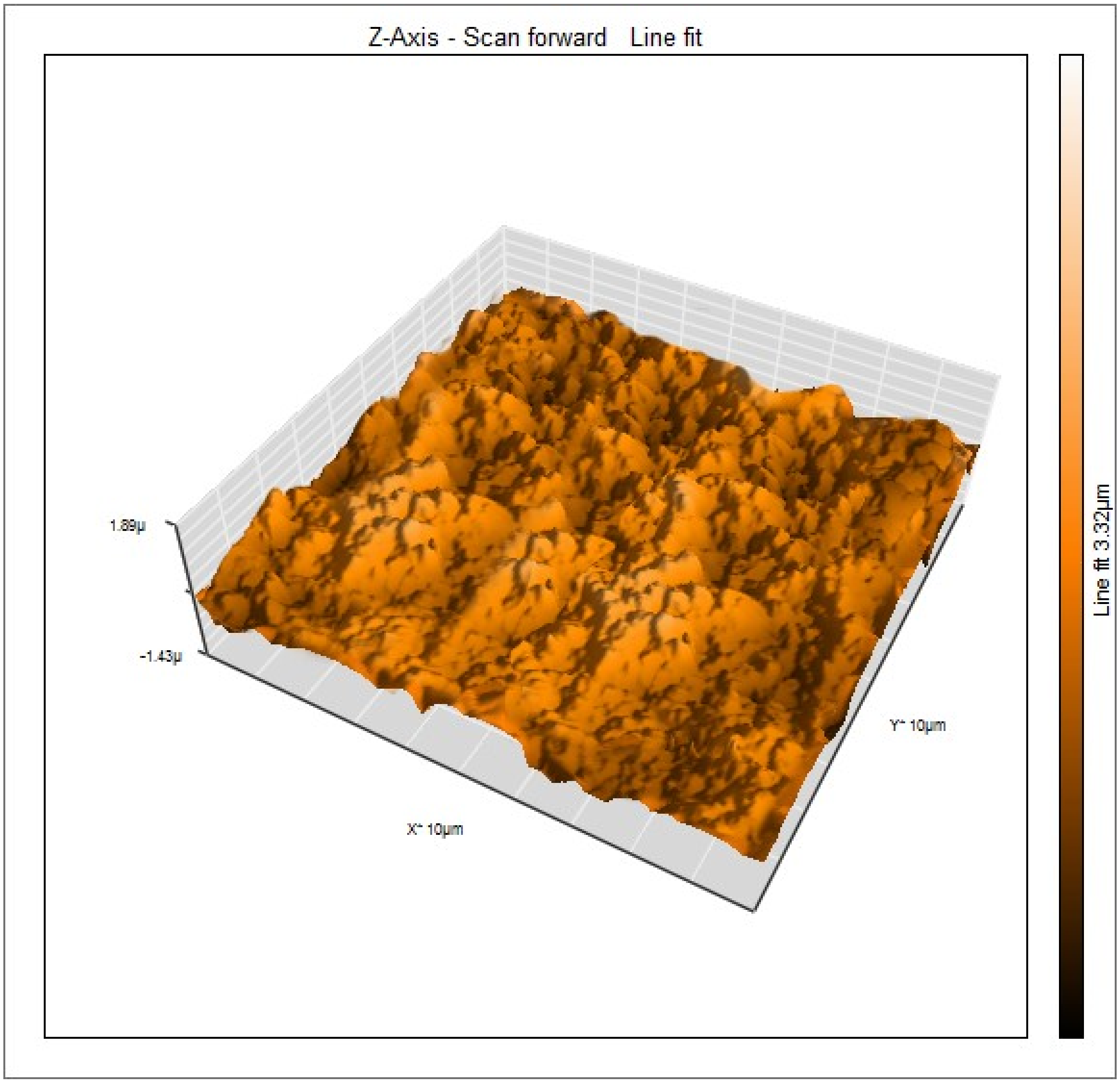

S18: 3-d image (1.0 M HCl + surfactant I)

Tool result

Name	Value
Area	100.8pm^2
Sa	180.67nm
Sq	238.33nm
Sy	2235.7nm
Sp	1145.1nm
Sv	-1090.6nm
Sm	-13.603fm

Store

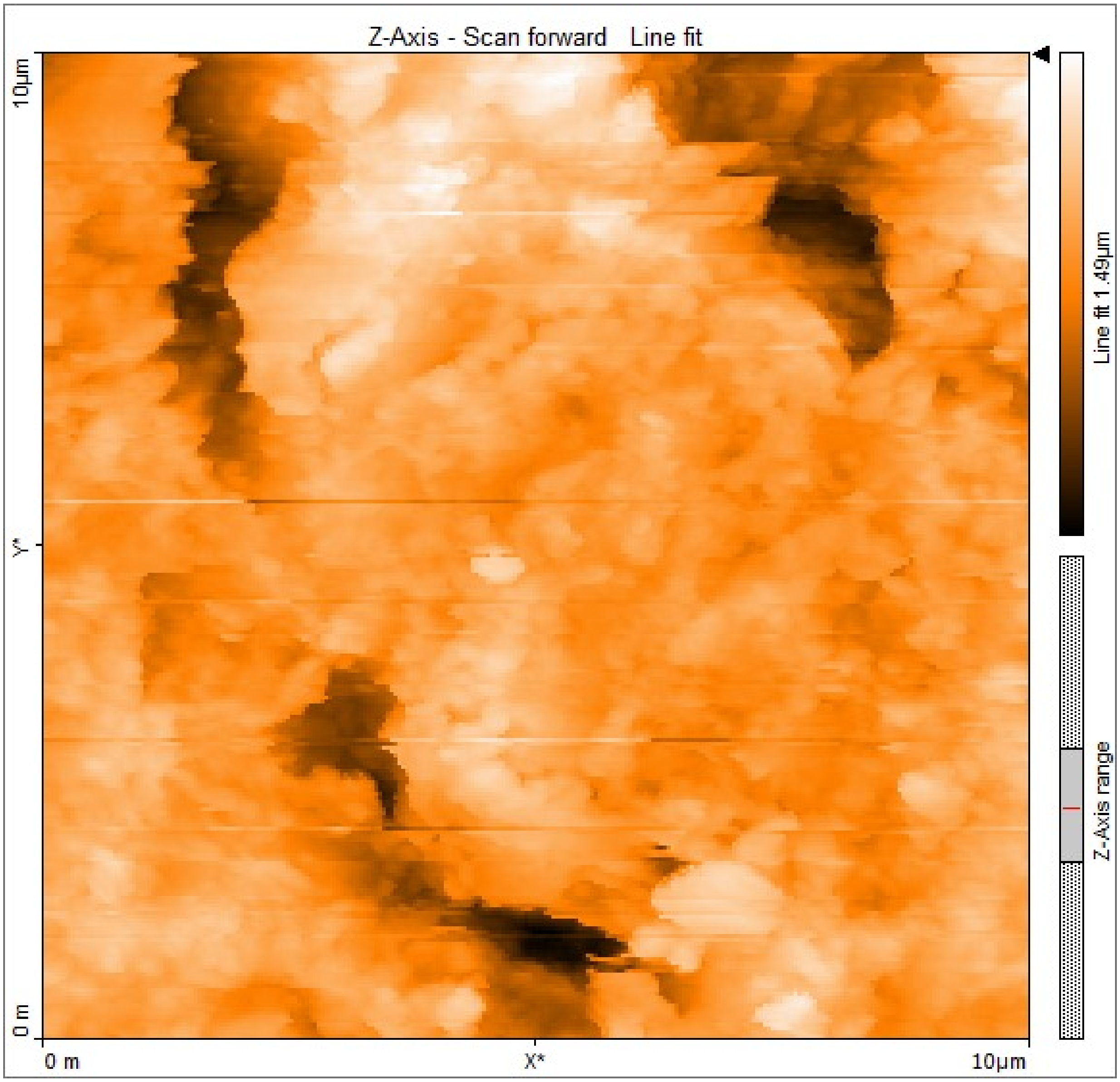

S19: 2-d image (1.0 M HCl + surfactant II)

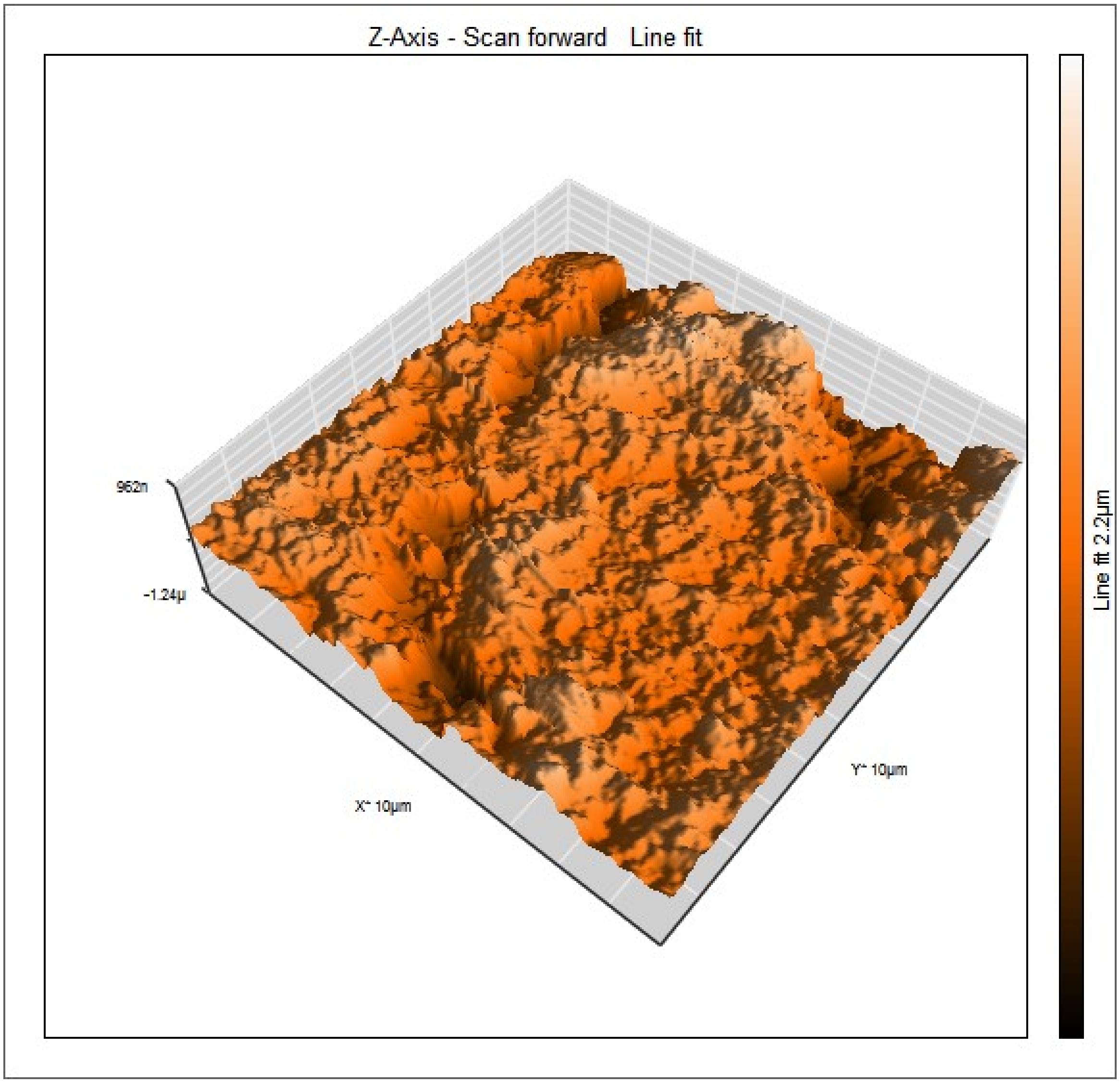

S20: 3-d image (1.0 M HCl + surfactant II)

Tool result

Name	Value
Area	100.8pm^2
Sa	164.18nm
Sq	210.89nm
Sy	1892.7nm
Sp	1332.2nm
Sv	-560.59nm
Sm	-13.269fm

Store

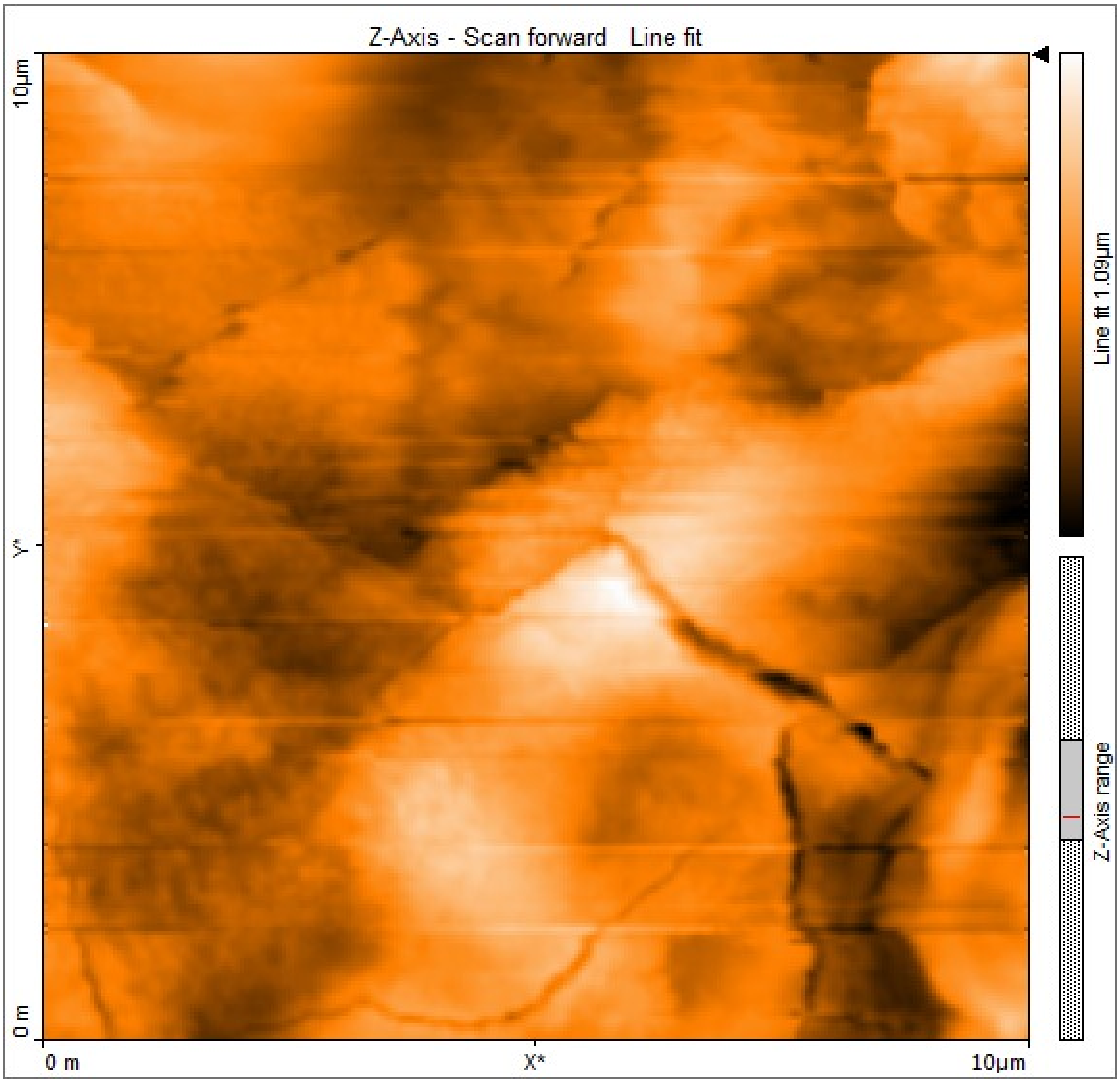

S21: 2-d image (1.0 M HCl + surfactant III)

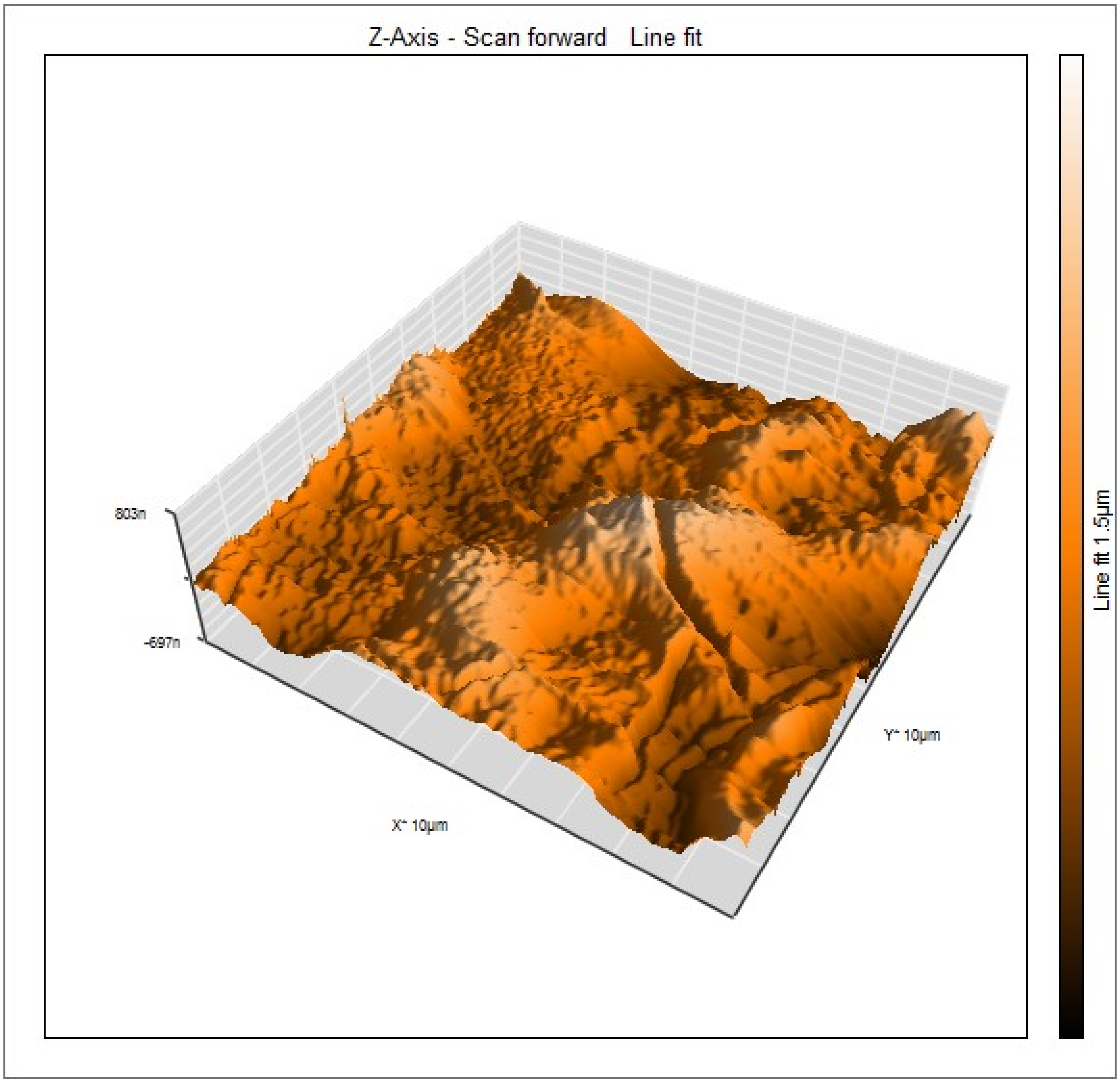

S22: 3-d image (1.0 M HCl + surfactant III)

Tool result

Name	Value
Area	100.8pm^2
Sa	133.22nm
Sq	163.94nm
Sy	1270.3nm
Sp	704.85nm
Sv	-565.41nm
Sm	-14.282fm

Store
